# TMBocelot: an omnibus statistical control model optimizing the TMB thresholds with systematic measurement errors

**DOI:** 10.3389/fimmu.2024.1514295

**Published:** 2025-01-20

**Authors:** Xin Lai, Shaoliang Wang, Xuanping Zhang, Xiaoyan Zhu, Yuqian Liu, Zhili Chang, Xiaonan Wang, Yang Shao, Jiayin Wang, Yixuan Wang

**Affiliations:** ^1^ School of Computer Science and Technology, Faculty of Electronics and Information Engineering, Xi’an Jiaotong University, Xi’an, Shaanxi, China; ^2^ Geneseeq Research Institute, Nanjing Geneseeq Technology Inc., Nanjing, Jiangsu, China; ^3^ School of Public Health, Nanjing Medical University, Nanjing, Jiangsu, China; ^4^ Department of Biomedical Engineering, College of Automation Engineering, Nanjing University of Aeronautics and Astronautics, Nanjing, China

**Keywords:** tumor mutation burden, immunotherapy endpoints, pairwise error control, positive-threshold optimization, Bayesian framework

## Abstract

Tumor mutation burden (TMB), defined as the number of somatic mutations of tumor DNA, is a well-recognized immunotherapy biomarker endorsed by regulatory agencies and pivotal in stratifying patients for clinical decision-making. However, measurement errors can compromise the accuracy of TMB assessments and the reliability of clinical outcomes, introducing bias into statistical inferences and adversely affecting TMB thresholds through cumulative and magnified effects. Given the unavoidable errors with current technologies, it is essential to adopt modeling methods to determine the optimal TMB-positive threshold. Therefore, we proposed a universal framework, TMBocelot, which accounts for pairwise measurement errors in clinical data to stabilize the determination of hierarchical thresholds. TMBocelot utilizes a Bayesian approach based on the stationarity principle of Markov chains to implement an enhanced error control mechanism, utilizing moderately informative priors. Simulations and retrospective data from 438 patients reveal that TMBocelot outperforms conventional methods in terms of accuracy, consistency of parameter estimations, and threshold determination. TMBocelot enables precise and reliable delineation of TMB-positive thresholds, facilitating the implementation of immunotherapy. The source code for TMBocelot is publicly available at https://github.com/YixuanWang1120/TMBocelot.

## Introduction

Immune checkpoint inhibitors (ICIs), exemplified by Programmed Cell Death-1 (PD-1)/Programmed Death-ligand 1 (PD-L1) inhibitors, have conferred significant clinical benefits across various cancer types (1–7). However, these benefits are not universal; only a subset of patients respond favorably to ICIs. Accurately identifying patients who are most likely to benefit from immunotherapy is crucial for optimizing treatment strategies and improving patient outcomes.

Tumor mutation burden (TMB), quantifies the number of somatic mutations per megabase of tumor DNA and has emerged as a key biomarker for predicting responses to ICIs. A higher TMB suggests a greater likelihood of producing neoantigens—novel proteins recognized as foreign by the immune system—which can enhance immune detection and elimination of cancer cells (11–13). Regulatory agencies worldwide have endorsed TMB as the sole guiding biomarker for pan-cancer immunotherapy applications (8–10). Determining the optimal TMB threshold that defines a “TMB-positive” patient is of paramount therapeutic importance for clinical decision-making (14, 15). An appropriate threshold assists clinicians in effectively screening potentially superior patients, ensuring effective treatment while avoiding unnecessary side effects in patients less likely to benefit. Optimizing this threshold is critical for maximizing therapeutic efficacy and personalizing cancer care.

Nevertheless, standardizing TMB thresholds is complicated by two significant computational challenges. First, the benefits of immunotherapy are multifaceted, encompassing measurable tumor shrinkage and prolonged survival (16). Effective patient selection should consider all relevant clinical outcomes. Integrating multiscale clinical endpoints—such as tumor response rates and survival times—into a single predictive model requires advanced statistical methods capable of handling different data types and relationships. Secondly, accurate measurement of TMB and assessment of clinical outcomes involve inherent uncertainties and potential errors (17–19). For TMB, factors such as tumor heterogeneity, variations in sequencing technologies, computational algorithm differences, and tumor purity can introduce inaccuracies (20–22). As a result, the measured TMB (labeled with $TMB^{*}$ ) is an approximation of the true value, expressed as $TMB^{*}=TMB+e_{1}$ , where $e_{1}$ represents measurement error may not conventionally adhere to parametric statistical distributions. Correspondingly, clinical endpoints paired with TMB, especially tumor response, also entail a high risk of measurement error. The assessment of objective tumor response is often based on imaging criteria like the Response Evaluation Criteria in Solid Tumors (RECIST 1.1) (21), determined by a single measurement of the maximum tumor diameter in the axial plane (29) and is categorized into different statuses. The precision of response labels can be affected by measurement precision, reader interpretation, image quality, and patient-specific factors (30, 31). These noises may likewise introduce error perturbations to endpoint observations, $R^{*}=R+e_{2}$ . Errors are transmitted and amplified to the patient screening stage with downstream estimation derivations, further destroying the consistency of inference and meddling with thresholds. The formula derivations and schematic diagrams in the **Supplementary Material** illustrate the impact of pairwise error in immunotherapy. These measurement errors can distort the true relationship between TMB and patient outcomes, leading to unreliable thresholds and potentially suboptimal treatment decisions. Ignoring or mischaracterizing these errors compromises the accuracy of statistical inferences and affects clinical decision-making (17, 23–27).

To effectively address the pairwise errors, we present TMBocelot—an Omnibus statistical Control model Optimizing the TMB Thresholds with systematic measurement errors. The presented methodology can adopt targeted methods to meet the requirements of the error specification, demonstrating the ability to effectively design a judicious pairwise error control mechanism for joint models that incorporate multiscale clinical endpoints. TMBocelot leverages a Bayesian statistical approach, utilizing the properties of Markov chains and incorporating moderately informative priors to model and correct measurement errors in both TMB assessments and clinical outcomes.

Our methodology provides tailored solutions adaptable to different cancer types, measurement error characteristics, and clinical scenarios. The simulations provided empirical evidence supporting our proficiency in accurate estimation and reliability in threshold determination. Additionally, we applied TMBocelot to 4 retrospective cohorts of non-small cell lung cancer (NSCLC) to demonstrate the performance. Results suggest that the proposed model can achieve more comprehensive and robust TMB thresholds, offering valuable insights to enhance the treatment of cancer patients. The code for TMBocelot is available at https://github.com/YixuanWang1120/TMBocelot.

## Materials and methods

In clinical practice, errors in data can arise in many forms, ranging from almost negligible in high-quality, expensive techniques to partially observable with the addition of further data. Therefore, it is essential to develop a general framework that can handle different types of errors across these various conditions. The methods described below are tailored to specific scenarios in this study.

### Modeling without measurement errors

First, we state a method that does not take into account any error. To accurately determine the TMB-positivity thresholds from multifaceted efficacy analyses, previous work (27, 28) has integrated two types of clinical outcomes: binary tumor response (e.g., responder or non-responder) and continuous time-to-event (TTE) endpoints (e.g., survival or progression-free survival). This integration accounts for the within-subject dependency between these two types of endpoints. For simplicity, we refer to this method as the Bayesian Naïve Method (Bayes-NM).

Specifically, for patient i , $R_{i}$ denotes the tumor response status ( $R_{i}=1,\;0$ for response and non-response, respectively), $Z_{i}$ denotes clinical covariates (e.g., age, gender, stage of cancer), and $TMB_{i}$ denotes the error-free biomarker. The tumor response $R_{i}$ depends on both the covariates $Z_{i}$ and $TMB_{i}$ . We model this using a logistic regression model, which relates the probability of response to these factors:


(1) $$\text{logit}(R_{i}|Z_{i},\;TMB_{i},\;b_{i};\;\theta )=\alpha_{z}^{\;T}Z_{i}+\alpha_{m}TMB_{i}+b_{i}$$

where θ represents a vector of unknown parameters.; $\alpha_{z}\;$ and $\alpha_{m}$ denotes regression coefficients for the covariates $Z_{i}$ and $TMB_{i}$ ; $b_{i}$ is a random effect for patient *i* (accounting for unmeasured individual variations).

Next, for time-to-event analysis, the event time $T_{i}$ represents the observed time until the occurrence of an event (e.g., tumor recurrence, progression, or death), which is taken as the minimum of the actual event time $U_{i}$ and the censoring time $C_{i}$ . Define the event indicator as ${\text{Δ}}_{i}=I(U_{i}\leq C_{i})$ , where I(·) is the indicator function that equals 1 if the event occurs and 0 if censored. For the time-to-event (TTE) data, we use the Cox proportional hazards (Cox-PH) model, which focuses on classifying patients by their survival risks:


(2) $$h_{i}(t|Z_{i},\;TMB_{i},\;b_{i};\;\theta )=h_{0}(t)\text{exp}(\beta_{z}^{\;T}Z_{i}+\beta_{m}TMB_{i}+b_{i})$$

where $h_{i}(t)$ describes the instantaneous risk for patient *i* at time *t*; $h_{0}(t)$ is known as the baseline hazard, typically modeled using the Weibull distribution; $\beta_{z}$ and $\beta_{m}$ is effect coefficients for the covariates $Z_{i}$ and $TMB_{i}$ ; the shared term $b_{i}$ is a random effect term accounting for correlation between event times and responses. The random effect $b_{i}$ is assumed to follow a normal distribution $N(0,\;\sigma_{b}^{2})$ , which represented the intra-subject correlation between event times and individual response.

The observed dataset for Bayes-NM is denoted as $D_{n}={\left\{R_{i},T_{i},\Delta_{i},Z_{i},TMB_{i}\right\}}_{i=1}^{n}$ . Multiscale endpoints (response and survival) can be jointly modeled by incorporating random effects and adjusting for the dependencies between the response probabilities and event times. Formally, the joint likelihood for the data is given by:


$$p(R_{i},\;T_{i},{\text{ Δ}}_{i},\;b_{i};\;\theta )=p(R_{i}|b_{i};\;\theta )\cdot p(T_{i},{\text{ Δ}}_{i}|b_{i};\;\theta )\cdot p(b_{i};\;\theta )$$

The joint log-likelihood function is then:


(3) $$\ell (\theta )=\sum_{i}\log\int p(R_{i}|b_{i};\;\theta )p(T_{i},{\text{ Δ}}_{i}|b_{i};\;\theta )p(b_{i};\;\theta )db_{i}$$

Inference about parameters θ is typically based on the maximization of this log-likelihood. Since the likelihood involves random effects, we use Markov Chain Monte Carlo (MCMC) sampling to estimate the posterior distribution of the parameters A.

To begin Bayesian inference, we must specify prior distributions for the unknown parameters. For the regression coefficients and variance components, we use non-informative priors as follows:


(4) $$\begin{array}{l} \alpha_{z},\;\alpha_{m},\;\beta_{z},\;\beta_{m}∼N(0,\;10^{2})\\ \lambda ∼gamma(0.001,\;0.001)\\ \sigma_{b}^{\;-2}∼gamma(0.001,\;0.001) \end{array}$$

These priors reflect weak prior knowledge and allow the data to predominantly drive the posterior estimates.

Once the priors are defined, we use MCMC to sample from the posterior distribution of the parameters. The parameters are estimated as the average of the posterior sample:


$$\hat{\theta }\approx \frac{1}{K}\sum_{k=1}^{K}\theta_{k}^{'}$$

where $\theta_{k}^{\prime }$ is the *k*-th sample from the posterior distribution and *K* is the number of MCMC iterations.

Equations 1, 2, and 4 comprise the core of Bayes-NM, assuming the absence of measurement errors, with further elaboration available in the **Supplementary Material**.

### Modeling under TMB error control

In practice, the measured TMB (i.e. $TMB^{*}$ ) is an approximation of the true TMB, due to inherent measurement errors, i.e., $TMB^{*}=TMB+e$ , where both TMB and e are unobserved potential variables and mutually independent. While external or internal validation sets can sometimes provide information on error characteristics, they may not always be available. This complexity in real-world data necessitates different strategies for handling errors. Below, we describe several methods for addressing these errors in the context of TMB measurement.

#### Corrected-score for normal TMB error

In certain cases, the error $e_{i}$ in the measurement of TMB follows a normal distribution with known variance $\sigma_{e}$ . To address this, we use the Corrected-Score Method (CSM), which is an estimation technique that adjusts for the measurement error in TMB (27). The CSM ensures that the first-order derivative of the likelihood (denoted as $\;{\text{Ψ}}_{c}^{*}$ ) is unbiased for the true score function, given the true TMB. This property can be written as:


$$\begin{array}{c} E\{{\text{Ψ}}_{c}^{*}\left(R_{i},\;T_{i},{\text{ Δ}}_{i},\;Z_{i},\;TMB_{i}^{*};\text{ Θ}\right)|TMB_{i}\}\\ =\text{Ψ}\left(R_{i},\;T_{i},{\text{ Δ}}_{i},\;Z_{i},\;TMB_{i}|\text{Θ}\right) \end{array}$$

This property means that the expected value of the corrected score is equal to the score based on the true TMB values. The method is conditionally unbiased, meaning it provides a reliable estimate of the parameters when the true TMB is known (32).

Despite its advantages, the CSM has limitations. It assumes that the measurement error eie_iei follows a normal distribution with known variance and cannot handle situations where there is misclassification in the clinical endpoints (e.g., incorrect tumor response classification). If such assumptions are not met, this approach may not perform optimally. In these cases, alternative methods like a non-parametric deconvolution approach (33) could be used to estimate the measurement error distribution.

#### Robust correction for unspecified TMB error

When there is no prior information about the error distribution, the CSM becomes impractical. To address this limitation, we expand upon the original Bayesian model by treating both TMB and the measurement error $\;e_{i}$ as random variables. This extension leads to the Bayesian Error Correction Method (Bayes-ECM), which does not rely on a normal distribution assumption for the measurement error. The Bayes-ECM models the measurement error and the true TMB using Dirichlet Process (DP) priors. Specifically:


$$f_{e}∼DP(M_{e},\;G_{e})$$

$$f_{TMB}∼DP(M_{TMB},\;G_{TMB})$$

where DP stands for Dirichlet Process, a distribution that allows for flexible modeling of uncertainty and is used here to model the uncertainty in the error distribution and the true TMB values (34). The parameters $M_{e}$ and $\;G_{e}$ define the base measure and concentration parameter for the error distribution, and similarly, $M_{TMB}$ and $\;G_{TMB}$ define those for the true TMB.

While the Dirichlet Process allows for robust modeling of measurement error and TMB, it yields a random, discontinuous distribution, making continuous density estimation challenging. To overcome this limitation, we convolve the Dirichlet Process with a continuous kernel or treat the Dirichlet Process as a mixing measure over parametric forms (35). Take ϕ as a typically finite-dimensional parameter space and let $f_{\varphi }$ be a continuous probability distribution function for each φ∈ϕ . Then $f_{e}(e)$ with $\varphi =(\mu ,\;\sigma^{2})$ may be:


$$f_{e}(e)=\int f_{\varphi }(e)dG(\varphi )$$

$$G∼DP(M_{e},\;G_{e})$$

$$f_{\varphi }(e)=N(e|\mu ,\;\sigma^{2})$$

This approach leads to a Gaussian Mixture Model (GMM), which can approximate any distribution with sufficient flexibility (35, 36).

The error distribution for TMB measurement can be expressed as a Gaussian Mixture:


(5) $$f_{e}(e)=\sum_{i}^{K}\pi_{i}N(\mu_{ei},\;\sigma_{e_{i}}^{\;2})$$

where K is the number of mixture components, determined by the data; $\pi_{i}$ is the mixing weight for each component, $\mu_{ei}$ and $\sigma_{ei}$ are the mean and variance of each Gaussian component, respectively.

Similarly, the true TMB values are modeled as:


(6) $$f_{TMB}(TMB)=\sum_{i}^{K}\pi_{i}N(\mu_{TMBi},\;\sigma_{TMB_{i}}^{\;2})$$

Where the mixture model allows flexibility in modeling both the true TMB and the measurement errors. The number of components *K* is determined by the data, providing a robust way to estimate the error distribution without needing external validation sets or prior assumptions. The **Supplementary Material** will discuss the Gaussian distribution-to-stochastic process conversion and parameter adjustments. Equations 1 and 2 describe the basic regression models for response and survival data, incorporating TMB. Equations 5, 6 describe the Gaussian Mixture Models for the measurement error and the true TMB.

In summary, the Bayes-ECM is a more general and robust approach compared to the CSM. It allows for the modeling of TMB measurement errors without relying on specific distributional assumptions and offers flexibility in cases where no external error information is available.

### Modeling under response error control

In addition to errors arising from TMB measurements, paired endpoints, such as clinical response, may also suffer from measurement inaccuracies. While errors in continuous TTE endpoints typically do not have a significant effect on the consistency of the analysis, response misclassification in discrete endpoints can substantially affect model accuracy and predictions. To address this, we propose the Bayesian Misclassification Correction Method (Bayes-MCM), which aims to account for misclassification errors in the observed response.

In this case, the observed $R_{i}\;$ is no longer an accurate reflection of the true tumor state but may be a misclassified version of the actual response $Y_{i}$ . To address this, we define the following misclassification parameters:


$$\eta =P(R_{i}=1|Y_{i}=1)$$

the probability of correctly classifying a true response (tumor shrinkage) as a positive response.


$$\delta =P(R_{i}=0|Y_{i}=0)$$

the probability of correctly classifying a true non-response (no tumor shrinkage) as a negative response.

To incorporate these parameters, the standard mixed-effects logistic regression model for the true response endpoint:


$$\text{logit}(Y_{i}|Z_{i},\;TMB_{i},\;b_{i};\;\theta )=\alpha_{z}^{T}Z_{i}+\alpha_{m}TMB_{i}+b_{i}$$

The corresponding model for the observed response is:


(7) $$\begin{array}{c} P(R_{i}=1|Z_{i},\;TMB_{i},\;b_{i};\;\theta )=\eta P(Y_{i}=1)\\ +(1-\delta )(1-P(Y_{i}=1)) \end{array}$$

where $P(Y_{i}=1)$ is derived from the logistic regression model.

Previous research (37) has demonstrated that without additional constraints or information, the misclassification parameters η and δ  are not practically identifiable. To overcome this limitation, we updated the Bayesian framework by incorporating informative priors and imposing constraints on these parameters. Specifically, the priors are modeled as Beta distributions:


(8) $$\begin{array}{l} \eta ∼Beta(\epsilon_{1},\;\epsilon_{2})\\ \delta ∼Beta(\epsilon_{3},\;\epsilon_{4}) \end{array}$$

where $\epsilon_{1},\epsilon_{2},\epsilon_{3},\epsilon_{4}$ are constants chosen based on prior knowledge or assumptions about misclassification rates. For example, η∼Beta(9, 1) assumes that the probability of correctly identifying a response is high, but not certain, with a mean of 0.9 and a 95% credible interval of [0.664, 0.997]. Conversely, δ∼Beta(72.5, 2.5) reflects a strong belief in the reliability of non-response classification, with a mean of 0.98 and a 95% credible interval of [0.917, 0.995]. These priors are constructed to reflect existing knowledge or reasonable assumptions about misclassification rates.

Furthermore, misclassification probabilities may vary across subpopulations, depending on factors such as tumor type, disease stage, or patient demographics. To address this, Bayes-MCM will incorporate hierarchical structures for *η* and δ, enabling the model to estimate separate parameters for different subgroups. This flexibility allows for tailored misclassification rates that reflect variations based on patient characteristics, such as prior treatments or response patterns.

The core of the Bayes-MCM includes Equations 2, 4, 7, and 8. Full mathematical details and derivations are provided in the **Supplementary Material**.

### Modeling under pairwise error control

While misclassification errors can occur in individual components (like TMB and response endpoints), real-world errors often involve both types of measurements simultaneously. The Bayesian Pairwise Error Correction Method (Bayes-PECM) extends the Bayes-MCM by simultaneously modeling both TMB errors and response misclassification.

In this comprehensive model, Equations 2, 4–8 jointly account for both sources of error. The model is flexible enough to adapt to the uncertainties in both TMB measurements and response misclassification, with details in the **Supplementary Material**.

### Framework for localizing TMB-positive thresholds

The Bayesian framework and pairwise error correction in TMBocelot enable a convergent and comprehensive estimate of ICI benefits for individual patients. By utilizing the joint likelihood to characterize a patient’s factual condition post-treatment and employing TMB as a biomarker, we used TMBcat to identify a stratification threshold based on the cut-off value with the minimum *p*-value (38, 39). Patients could be categorized into two categories for treatment prognosis comparison based on such a threshold.

**Figure 1:**
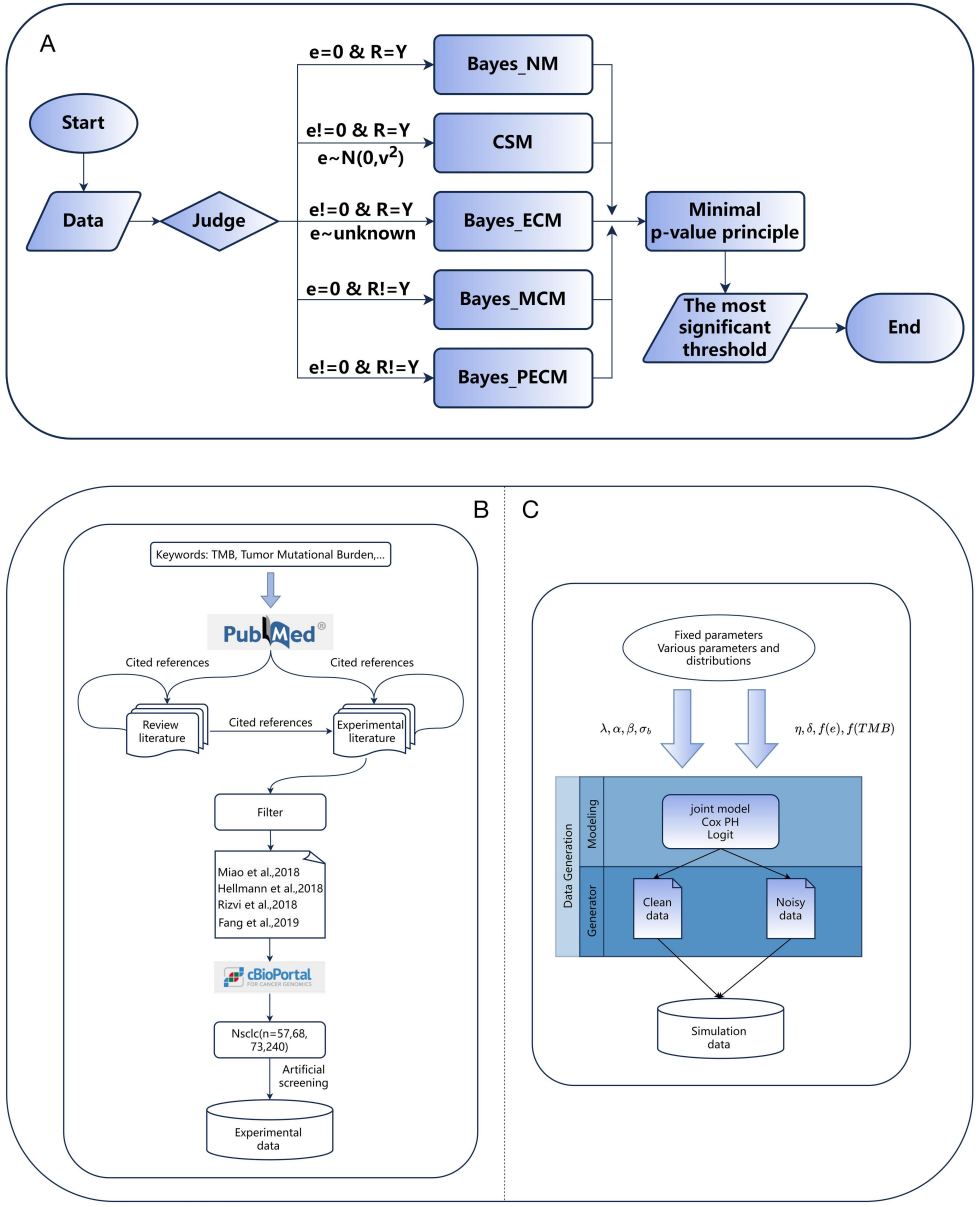
**(A)** The flow chart for the TMB threshold identification framework. **(B, C)** The process of generating simulation data and collecting experimental data.

We developed a robust framework for determining TMB-positive thresholds, ensuring broad applicability across diverse error scenarios. Depending on the specific context, the corresponding methods outlined above can be applied for parameter estimation, followed by the use of joint probabilities to identify precise TMB thresholds. The complete framework is given in pseudocode in **Algorithm 1** and **Figure 1A**. To facilitate adoption and reproducibility, we provide implementation resources. The source code for TMBocelot, including the full implementation of the Bayesian framework, pairwise error correction, and threshold determination methods, is publicly accessible at https://github.com/YixuanWang1120/TMBocelot. This open-access repository serves as a practical guide for researchers and clinicians. By making the codebase available, we aim to bridge the gap between technical complexity and practical usability, ensuring TMBocelot can be readily integrated into clinical decision-making workflows.

Algorithm 1 **Framework for localizing TMB-positive thresholds.**

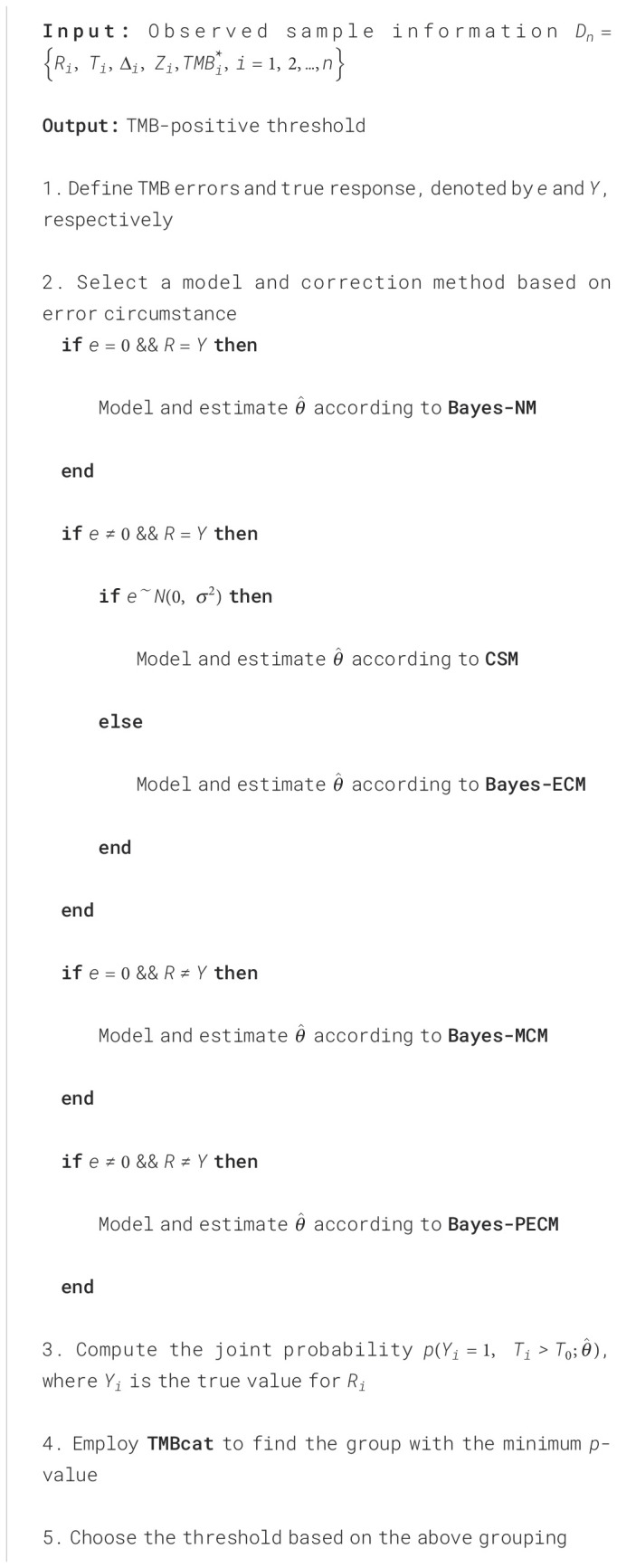

## Results

### Experimental patient cohorts

To validate the applicability of TMBocelot, we assembled four cohorts of 438 different patients from publicly available studies, encompassing 57, 68, 73, and 240 patients with NSCLC (26, 40–42). Our primary efficacy endpoints were tumor response and progression-free survival (PFS) evaluated according to the RECIST criteria. **Figure 1B** illustrates the data collection process. The specific patient information is listed in **Supplementary Table S1**.

The decision to focus exclusively on NSCLC cohorts was driven by the extensive availability of publicly accessible datasets for this cancer type and the well-established clinical relevance of TMB as a biomarker in NSCLC. Additionally, NSCLC represents a heterogeneous disease with varying response patterns to immune checkpoint inhibitors, making it a valuable model for evaluating the performance of TMBocelot.

Furthermore, we assessed the performance of TMBocelot in addressing various errors in oncology trials through a series of simulations. The simulations involved 200 individuals, each characterized by an individual-specific random effect $b_{i}$ across multiple endpoints. $b_{i}$ was drawn from a normal distribution with a mean of zero and variance $\sigma_{b}^{2}$ . Tumor response states were categorized as response ( $Y_{i}=1$ ) and non-response ( $Y_{i}=0$ ). $R_{i}=Y_{i}$ when misclassifications did not occur; otherwise, adjustments were made based on misclassification parameters η and δ . Actual response states were generated based on logistic probability, and patient event times were derived from a survival density following the Weibull distribution with a shape parameter of 1.0. Censoring time C was randomly generated from a uniform distribution U (0, 10).

We set $\alpha_{z}=-0.8,\;\alpha_{m}=0.4,\;\lambda =1.0,\;\beta_{z}=1.0,\;\beta_{m}=-0.4$ and $\sigma_{b}=0.5$ . $Z_{i}$ was generated from the uniform distribution U (0, 1). Various error scenarios were designed, including error-free, TMB errors only, endpoints misclassification only, and both. We used the corresponding approaches described above. In experiments focusing on TMB errors, we assigned different distributions to the true TMB and measurement errors to demonstrate the methods’ efficiency. We separately set η,δ to (0.70, 0.95) and (0.75, 0.98) for experiments considering response misclassification. Detailed settings can be found in the **Supplementary Table S2**. **Figure 1C** roughly depicts the generation of the simulation data.

### Pairwise error control enables accurate statistical inference

In the simulations, we report fitted values, average bias, standard deviation (SD), and standard error (SE) for each parameter. SD measures variability in the estimates across 500 simulations, while SE represents their average error.

**Table 1** summarizes parameter estimates, with extensive simulations detailed in **Supplementary Table S2**. These results highlight the robust performance of TMBocelot across various error scenarios. While TMBocelot performs slightly worse than the true-data estimator under measurement errors or misclassification, it significantly outperforms the naive estimator, which ignores such errors. This strongly supports TMBocelot’s effectiveness in handling diverse error types.

**Table 1:** Comparisons of bias and standard errors of estimators among various estimators.

Model and estimator	Coef	Fitted value	Average bias	SE	SD
TMB ~ Laplace (mean = 1, var = $1.5^{2}$ )					
True-data Bayesian estimator	λ	1.015	0.015	0.076	0.099
	β	1.011	0.011	0.193	0.215
	$\beta_{m}$	-0.404	-0.004	0.065	0.074
	α	-0.853	-0.053	0.328	0.304
	$\alpha_{m}$	0.426	0.026	0.116	0.116
	$\sigma_{b}$	0.476	-0.024	0.135	0.208
TMB with errors e ~ Normal (0, $1.0^{2}$ ) Naive estimator	λ	0.963	-0.037	0.070	0.082
	β	0.760	-0.240	0.177	0.199
	$\beta_{m}$	-0.270	0.130	0.051	0.054
	α	-0.637	0.163	0.303	0.292
	$\alpha_{m}$	0.286	-0.114	0.089	0.089
	$\sigma_{b}$	0.447	-0.053	0.123	0.204
TMB with errors e ~ Normal (0, $1.0^{2}$ ) Bayesian estimator	λ	1.030	0.030	0.089	0.101
	β	1.021	0.021	0.272	0.291
	$\beta_{m}$	-0.467	-0.067	0.133	0.124
	α	-0.783	0.017	0.388	0.331
	$\alpha_{m}$	0.443	0.043	0.186	0.159
	$\sigma_{b}$	0.462	-0.038	0.140	0.223
TMB with errors e ~ Normal (0, $1.0^{2}$ ) Corrected-score estimator	λ	1.001	0.001	0.056	0.092
	β	0.945	-0.055	0.152	0.233
	$\beta_{m}$	-0.385	0.015	0.054	0.093
	α	-0.783	0.017	0.316	0.349
	$\alpha_{m}$	0.384	-0.016	0.108	0.144
	$\sigma_{b}$	0.479	-0.021	0.044	0.207
TMB with errors e ~ Extreme (0, $1.0^{2}$ ) Naive estimator	λ	0.963	-0.037	0.070	0.086
	β	0.737	-0.263	0.177	0.195
	$\beta_{m}$	-0.264	0.136	0.050	0.057
	α	-0.653	0.147	0.306	0.303
	$\alpha_{m}$	0.293	-0.107	0.090	0.093
	$\sigma_{b}$	0.452	-0.048	0.125	0.198
TMB with errors e ~ Extreme (0, $1.0^{2}$ ) Bayesian estimator	λ	1.022	0.022	0.087	0.099
	β	0.992	-0.008	0.265	0.271
	$\beta_{m}$	-0.449	-0.049	0.127	0.128
	α	-0.792	0.008	0.384	0.341
	$\alpha_{m}$	0.447	0.047	0.183	0.149
	$\sigma_{b}$	0.460	-0.040	0.136	0.220
TMB with errors e ~ Extreme (0, $1.0^{2}$ ) Corrected-score estimator	λ	1.017	0.017	0.056	0.102
	β	0.933	-0.067	0.149	0.268
	$\beta_{m}$	-0.369	0.031	0.049	0.103
	α	-0.796	0.004	0.319	0.381
	$\alpha_{m}$	0.387	-0.013	0.112	0.161
	$\sigma_{b}$	0.528	0.028	0.045	0.203
Response with misclassification (η,δ)=(0.75,0.98) Naive estimator	λ	0.985	-0.015	0.045	0.054
	β	0.973	-0.027	0.115	0.123
	$\beta_{m}$	-0.395	0.005	0.039	0.042
	α	-1.250	-0.450	0.205	0.188
	$\alpha_{m}$	0.229	-0.171	0.064	0.063
	$\sigma_{b}$	0.429	-0.071	0.083	0.125
Response with misclassification (η,δ)=(0.75,0.98) Bayesian estimator	λ	1.015	0.015	0.052	0.066
	β	1.015	0.015	0.126	0.146
	$\beta_{m}$	-0.406	-0.006	0.042	0.047
	α	-0.877	-0.077	0.357	0.342
	$\alpha_{m}$	0.464	0.064	0.135	0.128
	$\sigma_{b}$	0.501	0.001	0.101	0.160
TMB with errors and response with misclassification e ~ Normal (0, $1.0^{2}$ ) (η,δ)=(0.75,0.98) Naive estimator	λ	0.949	-0.051	0.068	0.081
	β	0.721	-0.279	0.172	0.181
	$\beta_{m}$	-0.266	0.134	0.050	0.052
	α	-1.127	-0.327	0.312	0.262
	$\alpha_{m}$	0.158	-0.242	0.086	0.076
	$\sigma_{b}$	0.407	-0.093	0.119	0.189
TMB with errors and response with misclassification e ~ Normal (0, $1.0^{2}$ ) (η,δ)=(0.75,0.98) Bayesian estimator	λ	1.035	0.035	0.091	0.112
	β	1.025	0.025	0.277	0.287
	$\beta_{m}$	-0.465	-0.065	0.131	0.126
	α	-0.802	-0.002	0.599	0.389
	$\alpha_{m}$	0.449	0.049	0.297	0.180
	$\sigma_{b}$	0.483	-0.017	0.155	0.244
TMB with errors and response with misclassification e ~ Extreme (0, $1.0^{2}$ ) (η,δ)=(0.75,0.98) Naive estimator	λ	0.955	-0.045	0.069	0.081
	β	0.743	-0.257	0.173	0.194
	$\beta_{m}$	-0.263	0.137	0.049	0.052
	α	-1.124	-0.324	0.314	0.266
	$\alpha_{m}$	0.165	-0.235	0.086	0.081
	$\sigma_{b}$	0.434	-0.066	0.122	0.183
TMB with errors and response with misclassification e ~ Extreme (0, $1.0^{2}$ ) (η,δ)=(0.75,0.98) Bayesian estimator	λ	1.048	0.048	0.093	0.124
	β	1.021	0.021	0.272	0.305
	$\beta_{m}$	-0.452	-0.052	0.129	0.133
	α	-0.808	-0.008	0.602	0.397
	$\alpha_{m}$	0.434	0.034	0.289	0.179
	$\sigma_{b}$	0.508	0.008	0.157	0.260

Additionally, distinguishing between the corrected-score and Bayesian robust methods is crucial when considering only TMB errors. The results indicate: i) Regardless of the distribution of actual TMB, the corrected-score outperforms the Bayesian method when TMB errors follow a normal distribution with a standard deviation of 1.0. However, the Bayesian method performs better for errors with a larger deviation. The corrected-score may be more effective for minor errors, while the Bayesian robust method is preferable for more significant errors. ii) If TMB errors follow the extreme value distribution, the corrected-score’s performance deteriorates compared to the normal distribution, while the Bayesian method remains stable. This suggests that the corrected scoring method’s normality assumption may mis-specify the error distribution, especially in asymmetric distributions. Conversely, the Bayesian method demonstrates robustness concerning TMB error and truth value distribution. iii) The SE and SD for the Bayesian robust method exceeded those for the corrected-score method, attributed to a lack of *a priori* information. These limitations become more pronounced when accurately and robustly estimating the distribution of TMB actual values. The corrected-score is recommended for minor errors and a known variance; the Bayesian robust method is preferable for more significant errors or asymmetric distributions.

To evaluate the effect of errors on TMB thresholds and TMBocelot’s stability, we simulated treatment efficacy across different thresholds, considering 500 patients with actual TMBs positively correlated to favorable outcomes. The coefficients were set to $\;\alpha_{z}=-1.8,\;\alpha_{m}=0.4,\;\lambda =1.0,\;\beta_{z}=2.2,\;\beta_{m}=-0.4$ and $\sigma_{b}=0.5$ . TMB errors were generated by the normal distribution with a standard deviation of 1.0 and misclassification parameters η=0.75, δ=0.98 . We derived different thresholds for classifying patients and comparing the treatment efficacy of TMB-based subgroups.

**Figure 2:**
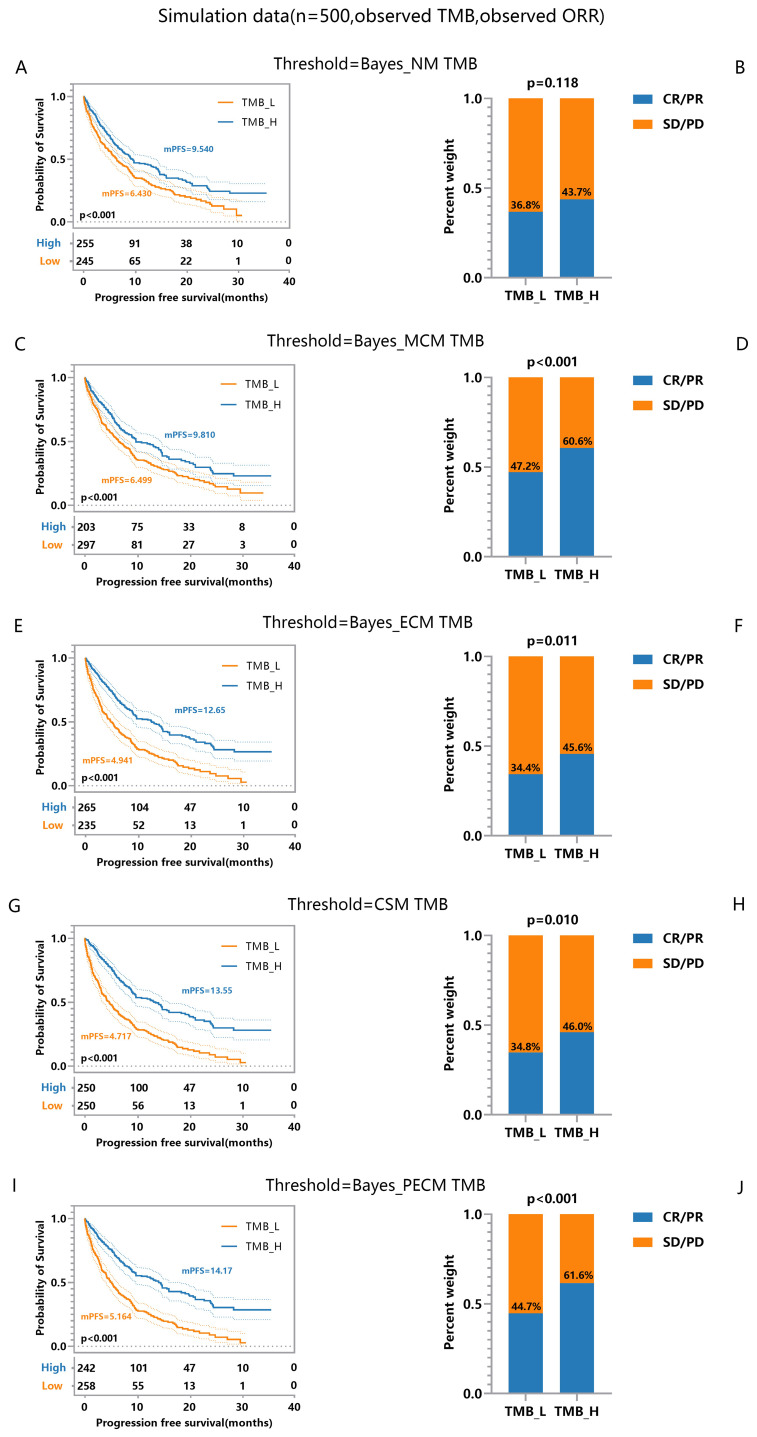
Efficacy Comparisons of Patients Grouped Based on Different TMB Thresholds. **(A, B)** Comparison of response and survival curves based on the threshold derived from Bayes-NM ignoring TMB errors and misclassification of response. **(C, D)** Comparison of response and survival curves based on the threshold derived from Bayes-MCM ignoring TMB errors. **(E, F)** Comparison of response and survival curves based on the threshold derived from Bayes-ECM ignoring misclassification of response. **(G, H)** Comparison of response and survival curves based on the threshold derived from Bayes-CSM ignoring misclassification of response. **(I, J)** Comparison of response and survival curves based on the threshold derived from Bayes-PECM considering both TMB errors and misclassification of response.

It is worth noting that the selection of $TMB_{i}$ and $R_{i}$ in the thresholding process and the efficacy comparison. When accounting for TMB errors or tumor response misclassification, we used the corresponding posterior estimates. For scenarios ignoring errors, the observed values were used. The comparison outcomes are depicted in **Figure 2**.

Significant efficacy disparities between TMB-low and TMB-high groups reflect the correlation between higher TMB and increased antitumor immunogenicity. Specifically, results show: i) Thresholds considering response misclassification result in greater disparities in tumor response (**Figure 2D**, *p* < 0.001) compared to the naive method (**Figure 2B**, *p* = 0.118), with similar survival curve disparities (**Figures 2A, C**). ii) The threshold considering TMB errors results in more significant disparities in both the survival curve and response (**Figures 2E-H**) compared to the threshold based on the naive method (**Figures 2A, B**) though tumor response disparities (**Figures 2F, H**, *p* = 0.011, 0.010) are slightly smaller than those considering only misclassification (**Figure 2D**, *p* < 0.001). iii) Thresholds considering both TMB errors and response misclassification yield the most significant disparities in survival and tumor response (**Figures 2I, J**) compared to all other methods (**Figures 2A–H**). These findings indicate that TMB errors impact survival and tumor response, while response misclassification primarily affects tumor response. The framework effectively establishes robust TMB thresholds, supporting clinical decision-making even with pairwise errors.

**Figure 3:**
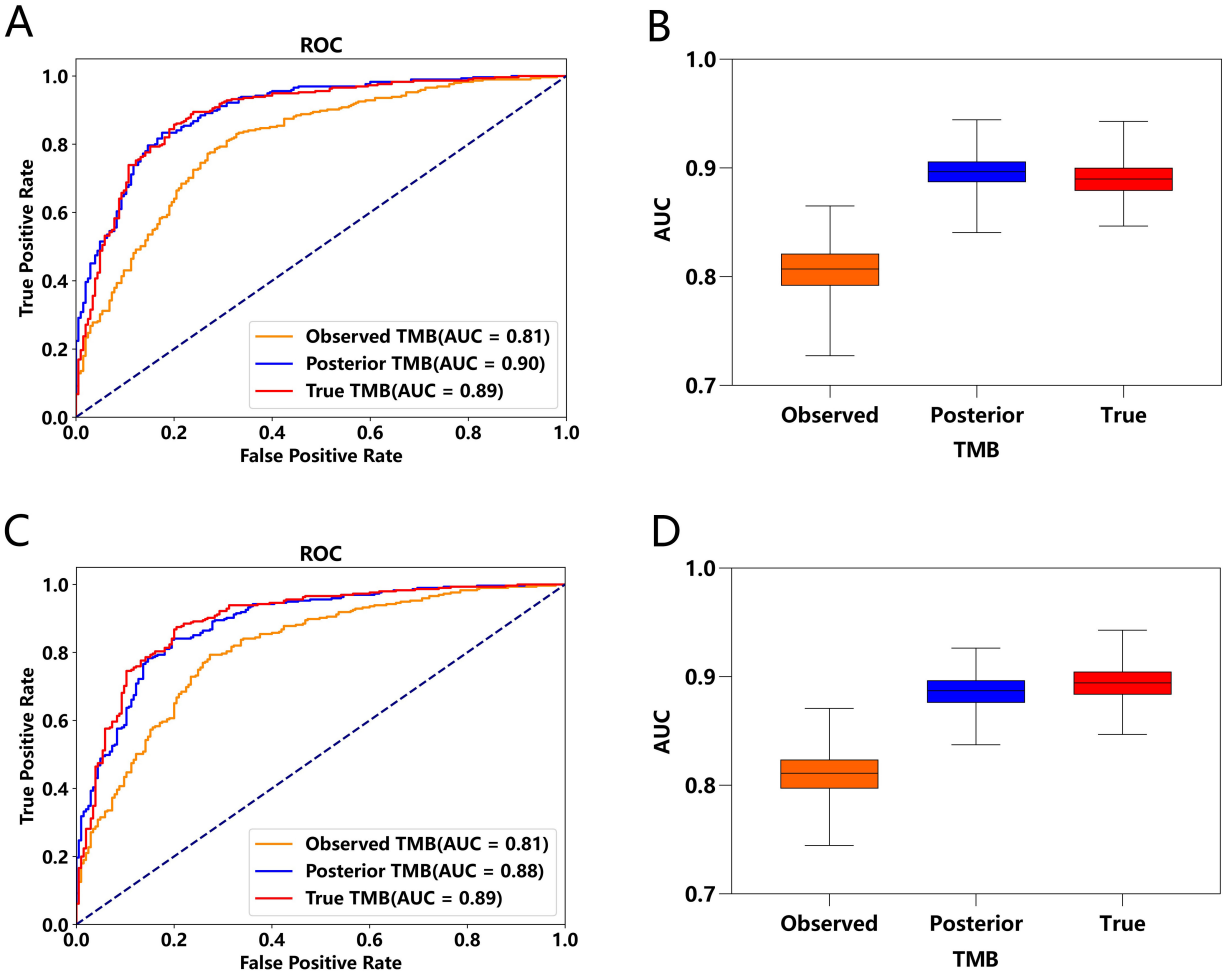
Comparison of ROC Curves and AUC under Observed TMB, Posterior TMB, and True TMB. **(A, C)** ROC curves for observed TMB, posterior TMB, and true TMB. **(B, D)** AUC values for observed TMB, posterior TMB, and true TMB.

To emphasize posterior TMB’s role in patient classification, we used accurate response labels. Logistic predictors based on observed, posterior, and true TMB generated ROC curves in **Figure 3**, where **Figures 3A**–**D** derive from the same data set, and the differences stem from the randomness of the MCMC algorithm. Results showed posterior and true TMB yielded similar AUC values, both significantly higher than those for observed TMB. Variability between posterior and true TMB AUCs arises from MCMC algorithm randomness and posterior TMB’s tendency toward maximum likelihood, which cannot fully match true TMB. While posterior TMB sometimes outperforms true TMB through parameter combination, it may also perform slightly worse. Overall, posterior TMB surpasses observed TMB in classification performance, underscoring its importance in patient stratification.

### Pairwise error control prompts robust efficacy stratification

**Figure 4:**
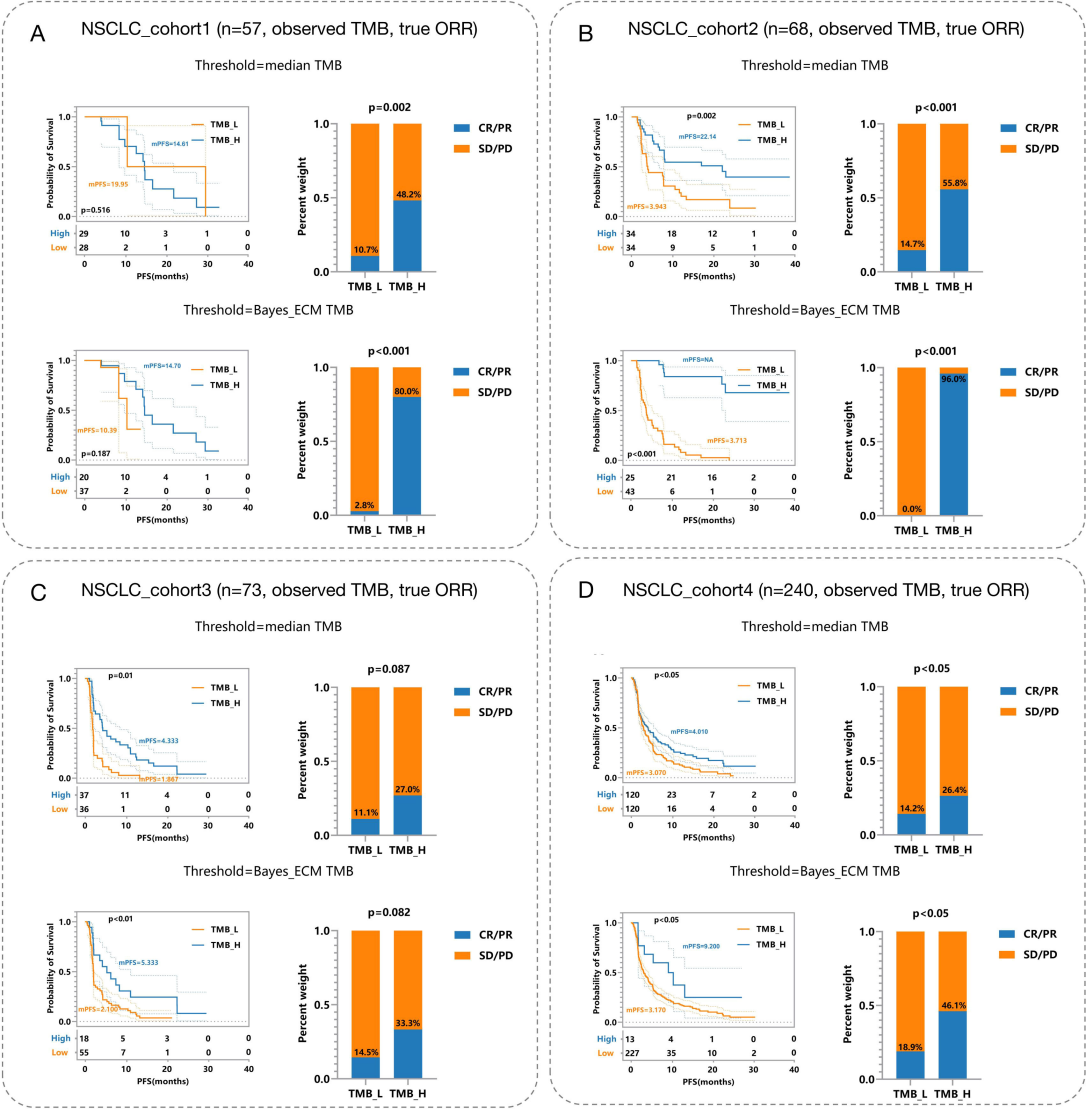
Efficacy Comparisons in NSCLC Patient Cohorts Based on Different TMB Thresholds. **(A)** NSCLC_cohort1 (n = 57): Observed TMB and true objective response rate (ORR). The top panels depict survival and response rates using median TMB thresholds, while the bottom panels utilize Bayes-ECM-derived thresholds. **(B)** NSCLC_cohort2 (n = 68): Similar setup as **(A)**, showcasing efficacy outcomes based on different TMB thresholds. **(C)** NSCLC_cohort3 (n = 73): Displays efficacy comparisons, similar to previous cohorts, highlighting survival curves and response rates. **(D)** NSCLC_cohort4 (n = 240): Largest cohort illustrating the impact of TMB thresholding on patient survival and response rates, analyzed through observed TMB and true ORR.

Incorporating error considerations, the multi-endpoint joint analysis marks a significant advancement by addressing real-world measurement challenges and surpassing previous studies. To validate TMBocelot, we applied the Bayes-ECM to each experimental cohort, considering TMB errors as recommended by the framework. Subsequently, we determined the TMB threshold by dividing patients into two subgroups using TMBcat and comparing it with the medians. The comparison outcomes are presented in **Figure 4**.

**Figure 5:**
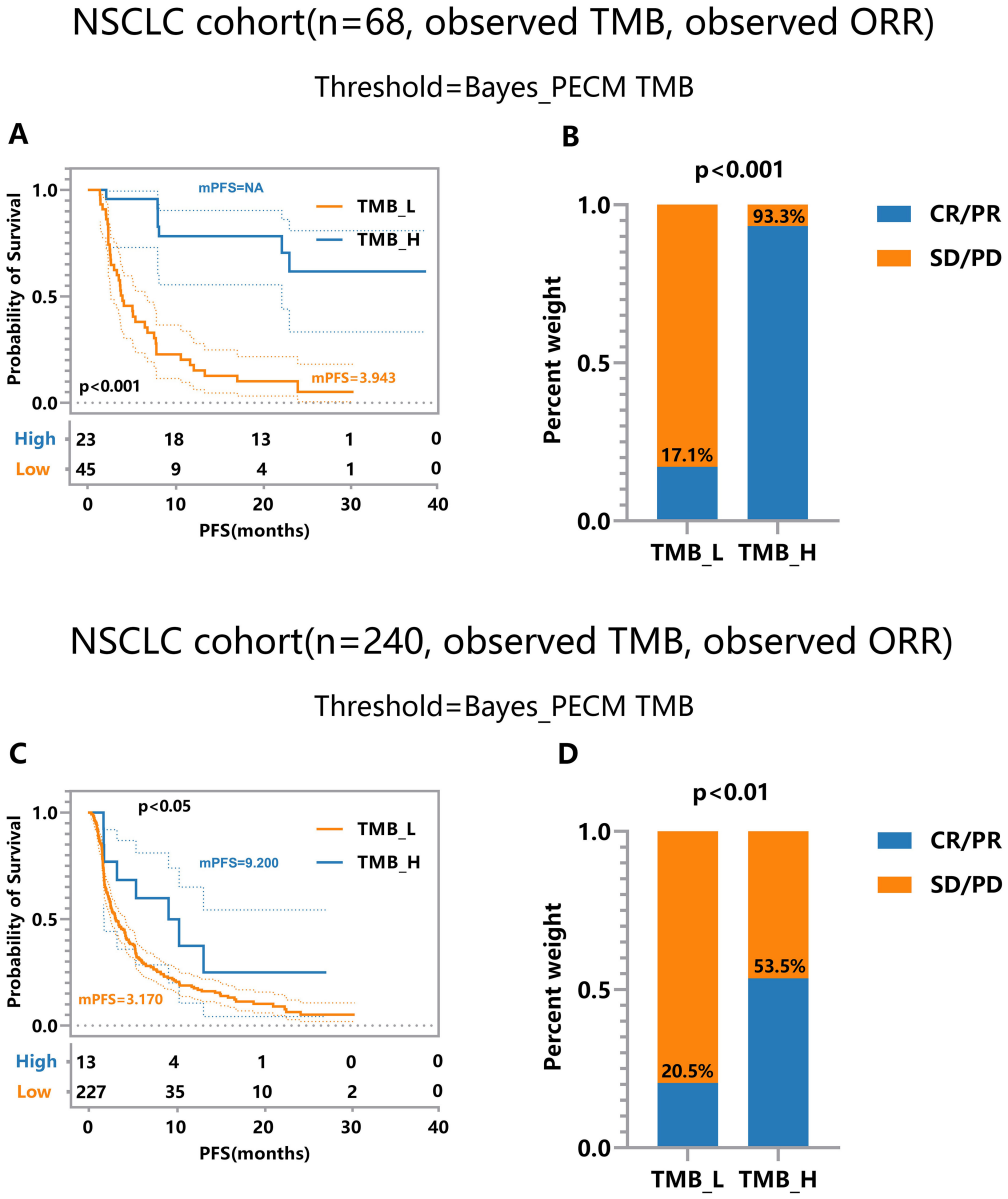
Efficacy Comparison of Patients Grouped Based on Different TMB Thresholds on Experimental NSCLC_cohort2&4 (n = 68, 240) under Observed TMB and Response with Perturbation. **(A, B)** Comparison of response and survival curves based on the threshold derived from Bayes-PECM for nsclc_68. **(C, D)** Comparison of response and survival curves based on the threshold derived from Bayes-PECM for nsclc_240.

The threshold calculated by the proposed method surpasses the medians in all cohorts. This superiority leads to more significant efficacy disparities, notably in nsclc_cohort1_57 (**Figure 4A**) and nsclc_cohort2_68 (**Figure 4B**), nsclc_cohort3_73 (**Figure 4C**) and nsclc_cohort4_240 (**Figure 4D**). Furthermore, to validate the proposed Bayes-PECM, we introduced artificial perturbations in tumor response for nsclc_cohort2_68 and nsclc_cohort4_240. This included a 25% error rate for patients with PR/CR and a 2% error rate for patients with PD/SD. The Bayes-PECM was tested on these two cohorts; the outcomes are depicted in **Figure 5**. **Figure 5** clearly shows pronounced efficacy disparities for nsclc_68 and nsclc_240, grouped by the Bayes-PECM-based threshold. Notably, the disparities in efficacy for experimental cohorts with added tumor response misclassification are similar to those achieved by experimental cohorts without misclassification, validating the effectiveness of the Bayes-PECM proposed in the framework.

A joint model with pairwise error control facilitates a more comprehensive and robust TMB subgrouping. This approach reveals more significant discrepancies between the efficacies of the TMB-low and TMB-high groups, showcasing the potential of the proposed framework in enhancing the precision and reliability of TMB subgroup classifications.

## Discussion

Tumor mutational burden (TMB) has recently garnered significant interest with its recognition by regulatory bodies as a biomarker, given the association of high TMB with improved responses to ICIs. However, defining clinically actionable TMB-positive thresholds remains contentious due to variations in evaluation metrics and the complexity of error sources, including TMB measurement errors and endpoint misclassification. Although recent studies have integrated multi-endpoints into TMB threshold analysis, error considerations have been relatively simplistic.

In response, we present a generalized framework, TMBocelot, capable of handling the complexity and diversity of real-world situations, accommodating various errors, including paired TMB errors and endpoint misclassification. Our simulations and applied experiments endorse this framework, enabling robust assessment of patient efficacy even amidst TMB errors and misclassified endpoints.

Additionally, this study yields valuable insights into the differential effects of errors on outcomes. While TMB errors exert a more pervasive influence on both survival and tumor response efficacy, endpoint misclassification primarily affects tumor response efficacy. These findings emphasize the need for tailored error correction methods. For instance, when measurement error is believed to be small and symmetrical, corrected-score methods leveraging auxiliary data or deconvolution approaches may suffice. In contrast, for complex or poorly understood error sources, Bayesian robust methods are recommended for error correction.

Despite its strengths, it is important to acknowledge the limitations of TMBocelot to provide a balanced perspective. First, the framework demands significant computational resources and large datasets, posing challenges for implementation in resource-constrained settings. High-dimensional Bayesian models, such as TMBocelot, require substantial processing power and expertise in Bayesian inference, potentially limiting their accessibility for smaller clinical or research institutions. Moreover, the reliance on high-quality datasets for accurate parameter estimation may reduce the framework’s applicability in scenarios where data availability or quality is limited. These resource-intensive requirements could hinder the widespread adoption of TMBocelot.

Another limitation lies in the scope of its validation, which was restricted to non-small cell lung cancer (NSCLC) cohorts. While the findings offer robust insights into the applicability of TMBocelot for NSCLC, their generalizability to other cancer types remains uncertain. Different cancer types may exhibit distinct biological characteristics and treatment response mechanisms, potentially leading to variations in TMB thresholds and response profiles. This limitation introduces potential biases and underscores the need for further validation across diverse tumor types. Expanding the framework’s application to a broader range of cancers would enhance its generalizability, enabling the development of cancer-specific refinements and ensuring its broader relevance.

## Conclusion

Measurement error is an unavoidable challenge in practical applications. However, the current approach to analyzing TMB thresholds tends to oversimplify error considerations. Our study is grounded in real-world scenarios and systematically accounts for various error scenarios to optimize the positive threshold. Theoretically, our method can result in a more comprehensive and robust TMB threshold. From the simulation and experimental results, we reasonably conclude that 1) our proposed joint model with the parameter estimation procedure can more robustly assess patient efficacy even under the interference of TMB errors and endpoints misclassification. 2) The error scenarios are complex and diverse, and we recommend choosing the scheme in the generalized framework according to the actual situation. 3) The TMB-positive threshold derived from multi-endpoint joint analysis considering errors can classify patients into two groups with more apparently stratified efficacy. Our model is applicable to clinical datasets with multiple endpoints and has the potential to significantly enhance physicians’ decision-making processes in clinical practice.

## Data Availability

The original contributions presented in the study are included in the article/**Supplementary Material**. Further inquiries can be directed to the corresponding authors.

## Supplementary material

Supplementary Table S1 The specific patient information in this study.

Supplementary Table S2 The extensive parameter estimates in simulations.

Supplementary Material The technical appendices for TMBocelot.
