# Supplementary material for: TMBocelot: an omnibus statistical control model optimizing the TMB thresholds with systematic measurement errors
Source: Front Immunol. 2025 Jan 20;15:1514295. doi: 10.3389/fimmu.2024.1514295 (PMC11788372; doi:10.3389/fimmu.2024.1514295)
Supplement: Supplementary Table S2 — The extensive parameter estimates in simulations. [file Table2.docx]

**Supplementary Table S2:** Comparisons of bias and standard errors of estimators among various estimators with varying error scenarios.

| **Model and estimator** | **Coef** | **Fitted value** | **Average bias** | **SE** | **SD** |
| --- | --- | --- | --- | --- | --- |
| TMB ~ Normal (mean = 1, var = ${1.5}^{2}$) | | | | | |
| True-data  Bayesian estimator | $\lambda$ | 1.005 | 0.005 | 0.048 | 0.058 |
|  | $\beta$ | 1.008 | 0.008 | 0.120 | 0.137 |
|  | $\beta_{m}$ | -0.403 | -0.003 | 0.040 | 0.043 |
|  | $\alpha$ | -0.801 | -0.001 | 0.201 | 0.206 |
|  | $\alpha_{m}$ | 0.407 | 0.007 | 0.067 | 0.064 |
|  | $\sigma_{b}$ | 0.489 | -0.011 | 0.088 | 0.133 |
| TMB with errors  e ~ Normal (0, ${1.0}^{2}$)  Naive estimator | $\lambda$ | 0.963 | -0.037 | 0.070 | 0.083 |
|  | $\beta$ | 0.749 | -0.251 | 0.175 | 0.184 |
|  | $\beta_{m}$ | -0.274 | 0.126 | 0.050 | 0.054 |
|  | $\alpha$ | -0.650 | 0.150 | 0.303 | 0.303 |
|  | $\alpha_{m}$ | 0.290 | -0.110 | 0.087 | 0.084 |
|  | $\sigma_{b}$ | 0.440 | -0.060 | 0.124 | 0.187 |
| TMB with errors  e ~ Normal (0, ${1.0}^{2}$)  Bayesian estimator | $\lambda$ | 1.020 | 0.020 | 0.088 | 0.097 |
|  | $\beta$ | 1.023 | 0.023 | 0.285 | 0.294 |
|  | $\beta_{m}$ | -0.493 | -0.093 | 0.145 | 0.120 |
|  | $\alpha$ | -0.789 | 0.011 | 0.396 | 0.346 |
|  | $\alpha_{m}$ | 0.473 | 0.073 | 0.198 | 0.149 |
|  | $\sigma_{b}$ | 0.427 | -0.073 | 0.137 | 0.214 |
| TMB with errors  e ~ Normal (0, ${1.0}^{2}$)  Corrected score estimator | $\lambda$ | 1.009 | 0.009 | 0.057 | 0.105 |
|  | $\beta$ | 0.972 | -0.028 | 0.152 | 0.249 |
|  | $\beta_{m}$ | -0.390 | 0.010 | 0.053 | 0.100 |
|  | $\alpha$ | -0.756 | 0.044 | 0.313 | 0.353 |
|  | $\alpha_{m}$ | 0.355 | -0.045 | 0.105 | 0.139 |
|  | $\sigma_{b}$ | 0.495 | -0.005 | 0.044 | 0.207 |
| TMB with errors  e ~ Normal (0, ${1.5}^{2}$)  Naive estimator | $\lambda$ | 0.928 | -0.072 | 0.067 | 0.085 |
|  | $\beta$ | 0.600 | -0.400 | 0.169 | 0.196 |
|  | $\beta_{m}$ | -0.198 | 0.202 | 0.042 | 0.047 |
|  | $\alpha$ | -0.534 | 0.266 | 0.292 | 0.278 |
|  | $\alpha_{m}$ | 0.212 | -0.188 | 0.073 | 0.076 |
|  | $\sigma_{b}$ | 0.423 | -0.077 | 0.121 | 0.197 |
| TMB with errors  e ~ Normal (0, ${1.5}^{2}$)  Bayesian estimator | $\lambda$ | 1.015 | 0.015 | 0.091 | 0.103 |
|  | $\beta$ | 0.992 | -0.008 | 0.299 | 0.314 |
|  | $\beta_{m}$ | -0.467 | -0.067 | 0.157 | 0.138 |
|  | $\alpha$ | -0.744 | 0.056 | 0.409 | 0.359 |
|  | $\alpha_{m}$ | 0.429 | 0.029 | 0.213 | 0.168 |
|  | $\sigma_{b}$ | 0.428 | -0.072 | 0.136 | 0.222 |
| TMB with errors  e ~ Normal (0, ${1.5}^{2}$)  Corrected score estimator | $\lambda$ | 0.948 | -0.052 | 0.055 | 0.088 |
|  | $\beta$ | 0.761 | -0.239 | 0.158 | 0.275 |
|  | $\beta_{m}$ | -0.276 | 0.124 | 0.053 | 0.127 |
|  | $\alpha$ | -0.834 | -0.034 | 0.328 | 0.376 |
|  | $\alpha_{m}$ | 0.417 | 0.017 | 0.103 | 0.158 |
|  | $\sigma_{b}$ | 0.414 | -0.086 | 0.042 | 0.168 |
| TMB with errors  e ~ Laplace (0, ${1.0}^{2}$)  Naive estimator | $\lambda$ | 0.956 | -0.044 | 0.070 | 0.082 |
|  | $\beta$ | 0.751 | -0.249 | 0.176 | 0.202 |
|  | $\beta_{m}$ | -0.273 | 0.127 | 0.050 | 0.055 |
|  | $\alpha$ | -0.644 | 0.156 | 0.304 | 0.284 |
|  | $\alpha_{m}$ | 0.292 | -0.108 | 0.088 | 0.090 |
|  | $\sigma_{b}$ | 0.431 | -0.069 | 0.125 | 0.198 |
| TMB with errors  e ~ Laplace (0, ${1.0}^{2}$)  Bayesian estimator | $\lambda$ | 1.037 | 0.037 | 0.091 | 0.107 |
|  | $\beta$ | 1.053 | 0.053 | 0.291 | 0.278 |
|  | $\beta_{m}$ | -0.494 | -0.094 | 0.146 | 0.124 |
|  | $\alpha$ | -0.804 | -0.004 | 0.405 | 0.350 |
|  | $\alpha_{m}$ | 0.487 | 0.087 | 0.202 | 0.151 |
|  | $\sigma_{b}$ | 0.477 | -0.023 | 0.477 | 0.222 |
| TMB with errors  e ~ Laplace (0, ${1.0}^{2}$)  Corrected score estimator | $\lambda$ | 0.999 | -0.001 | 0.056 | 0.094 |
|  | $\beta$ | 0.945 | 0.055 | 0.151 | 0.250 |
|  | $\beta_{m}$ | -0.376 | 0.024 | 0.051 | 0.096 |
|  | $\alpha$ | -0.811 | -0.011 | 0.315 | 0.341 |
|  | $\alpha_{m}$ | 0.391 | -0.009 | 0.105 | 0.138 |
|  | $\sigma_{b}$ | 0.499 | -0.001 | 0.045 | 0.196 |
| TMB with errors  e ~ Laplace (0, ${1.5}^{2}$)  Naive estimator | $\lambda$ | 0.925 | -0.075 | 0.067 | 0.079 |
|  | $\beta$ | 0.607 | -0.393 | 0.168 | 0.181 |
|  | $\beta_{m}$ | -0.198 | 0.202 | 0.041 | 0.048 |
|  | $\alpha$ | -0.539 | 0.261 | 0.292 | 0.271 |
|  | $\alpha_{m}$ | 0.218 | -0.182 | 0.074 | 0.078 |
|  | $\sigma_{b}$ | 0.413 | -0.087 | 0.120 | 0.186 |
| TMB with errors  e ~ Laplace (0, ${1.5}^{2}$)  Bayesian estimator | $\lambda$ | 1.017 | 0.017 | 0.090 | 0.109 |
|  | $\beta$ | 0.961 | -0.039 | 0.288 | 0.307 |
|  | $\beta_{m}$ | -0.454 | -0.054 | 0.149 | 0.155 |
|  | $\alpha$ | -0.730 | 0.070 | 0.405 | 0.339 |
|  | $\alpha_{m}$ | 0.436 | 0.036 | 0.213 | 0.171 |
|  | $\sigma_{b}$ | 0.440 | -0.06 | 0.138 | 0.224 |
| TMB with errors  e ~ Laplace (0, ${1.5}^{2}$)  Corrected score estimator | $\lambda$ | 0.960 | -0.040 | 0.054 | 0.090 |
|  | $\beta$ | 0.787 | -0.213 | 0.148 | 0.280 |
|  | $\beta_{m}$ | -0.284 | 0.116 | 0.044 | 0.123 |
|  | $\alpha$ | -0.819 | -0.019 | 0.342 | 0.402 |
|  | $\alpha_{m}$ | 0.414 | 0.014 | 0.148 | 0.179 |
|  | $\sigma_{b}$ | 0.446 | -0.054 | 0.044 | 0.181 |
| TMB with errors  e ~ Extreme (0, ${1.0}^{2}$)  Naive estimator | $\lambda$ | 0.953 | -0.047 | 0.070 | 0.084 |
|  | $\beta$ | 0.740 | -0.260 | 0.178 | 0.195 |
|  | $\beta_{m}$ | -0.265 | 0.135 | 0.046 | 0.055 |
|  | $\alpha$ | -0.632 | 0.168 | 0.303 | 0.290 |
|  | $\alpha_{m}$ | 0.290 | -0.110 | 0.088 | 0.091 |
|  | $\sigma_{b}$ | 0.451 | -0.049 | 0.127 | 0.194 |
| TMB with errors  e ~ Extreme (0, ${1.0}^{2}$)  Bayesian estimator | $\lambda$ | 1.040 | 0.040 | 0.091 | 0.101 |
|  | $\beta$ | 1.053 | 0.053 | 0.290 | 0.299 |
|  | $\beta_{m}$ | -0.512 | -0.112 | 0.150 | 0.130 |
|  | $\alpha$ | -0.801 | -0.001 | 0.401 | 0.341 |
|  | $\alpha_{m}$ | 0.491 | 0.091 | 0.204 | 0.152 |
|  | $\sigma_{b}$ | 0.451 | -0.049 | 0.139 | 0.223 |
| TMB with errors  e ~ Extreme (0, ${1.0}^{2}$)  Corrected score estimator | $\lambda$ | 1.006 | 0.006 | 0.056 | 0.101 |
|  | $\beta$ | 0.908 | -0.092 | 0.149 | 0.255 |
|  | $\beta_{m}$ | -0.367 | 0.033 | 0.048 | 0.107 |
|  | $\alpha$ | -0.792 | 0.008 | 0.315 | 0.358 |
|  | $\alpha_{m}$ | 0.384 | -0.016 | 0.105 | 0.143 |
|  | $\sigma_{b}$ | 0.516 | 0.016 | 0.045 | 0.206 |
| TMB with errors  e ~ Extreme (0, ${1.5}^{2}$)  Naive estimator | $\lambda$ | 0.928 | -0.072 | 0.067 | 0.082 |
|  | $\beta$ | 0.596 | -0.404 | 0.167 | 0.190 |
|  | $\beta_{m}$ | -0.185 | 0.215 | 0.040 | 0.046 |
|  | $\alpha$ | -0.526 | 0.274 | 0.293 | 0.263 |
|  | $\alpha_{m}$ | 0.215 | -0.185 | 0.074 | 0.079 |
|  | $\sigma_{b}$ | 0.427 | -0.073 | 0.119 | 0.193 |
| TMB with errors  e ~ Extreme (0, ${1.5}^{2}$)  Bayesian estimator | $\lambda$ | 1.026 | 0.026 | 0.094 | 0.103 |
|  | $\beta$ | 0.999 | -0.001 | 0.295 | 0.320 |
|  | $\beta_{m}$ | -0.453 | -0.053 | 0.153 | 0.150 |
|  | $\alpha$ | -0.783 | 0.017 | 0.415 | 0.358 |
|  | $\alpha_{m}$ | 0.437 | 0.037 | 0.214 | 0.169 |
|  | $\sigma_{b}$ | 0.466 | -0.034 | 0.141 | 0.230 |
| TMB with errors  e ~ Extreme (0, ${1.5}^{2}$)  Corrected score estimator | $\lambda$ | 0.965 | -0.035 | 0.054 | 0.103 |
|  | $\beta$ | 0.756 | -0.244 | 0.144 | 0.304 |
|  | $\beta_{m}$ | -0.268 | 0.132 | 0.039 | 0.127 |
|  | $\alpha$ | -0.822 | -0.022 | 0.357 | 0.402 |
|  | $\alpha_{m}$ | 0.391 | -0.009 | 0.123 | 0.175 |
|  | $\sigma_{b}$ | 0.463 | -0.037 | 0.043 | 0.198 |
| Response with misclassification  $\left( \eta,\delta\right)=\left( 0.70,0.95 \right)$  Naive estimator | $\lambda$ | 0.976 | -0.024 | 0.045 | 0.053 |
|  | $\beta$ | 0.966 | -0.034 | 0.114 | 0.126 |
|  | $\beta_{m}$ | -0.392 | 0.008 | 0.038 | 0.041 |
|  | $\alpha$ | -1.240 | -0.440 | 0.203 | 0.185 |
|  | $\alpha_{m}$ | 0.198 | -0.202 | 0.061 | 0.061 |
|  | $\sigma_{b}$ | 0.413 | -0.087 | 0.082 | 0.130 |
| Response with misclassification  $\left( \eta,\delta\right)=\left( 0.70,0.95 \right)$  Bayesian estimator | $\lambda$ | 1.006 | 0.006 | 0.051 | 0.067 |
|  | $\beta$ | 1.005 | 0.005 | 0.124 | 0.147 |
|  | $\beta_{m}$ | -0.402 | -0.002 | 0.040 | 0.046 |
|  | $\alpha$ | -0.816 | -0.016 | 0.350 | 0.315 |
|  | $\alpha_{m}$ | 0.393 | -0.007 | 0.116 | 0.107 |
|  | $\sigma_{b}$ | 0.487 | -0.013 | 0.097 | 0.163 |
|  | $\eta$ | 0.728 | 0.028 | 0.044 | 0.025 |
|  | $\delta$ | 0.965 | 0.015 | 0.020 | 0.005 |
| Response with misclassification  $\left( \eta,\delta\right)=\left( 0.75,0.98 \right)$  Naive estimator | $\lambda$ | 0.986 | -0.014 | 0.045 | 0.054 |
|  | $\beta$ | 0.968 | -0.032 | 0.115 | 0.130 |
|  | $\beta_{m}$ | -0.395 | 0.005 | 0.039 | 0.040 |
|  | $\alpha$ | -1.255 | -0.455 | 0.206 | 0.183 |
|  | $\alpha_{m}$ | 0.023 | -0.377 | 0.063 | 0.062 |
|  | $\sigma_{b}$ | 0.424 | -0.076 | 0.082 | 0.137 |
| Response with misclassification  $\left( \eta,\delta\right)=\left( 0.75,0.98 \right)$  Bayesian estimator | $\lambda$ | 1.015 | 0.015 | 0.052 | 0.069 |
|  | $\beta$ | 1.022 | 0.022 | 0.127 | 0.147 |
|  | $\beta_{m}$ | -0.406 | -0.006 | 0.041 | 0.046 |
|  | $\alpha$ | -0.887 | -0.087 | 0.356 | 0.327 |
|  | $\alpha_{m}$ | 0.460 | 0.060 | 0.124 | 0.119 |
|  | $\sigma_{b}$ | 0.512 | 0.012 | 0.102 | 0.164 |
|  | $\eta$ | 0.733 | -0.017 | 0.043 | 0.024 |
|  | $\delta$ | 0.964 | -0.016 | 0.020 | 0.005 |
| TMB with errors and response with misclassification  e ~ Normal (0, ${1.0}^{2}$)  $\left( \eta,\delta\right)=\left( 0.70,0.95 \right)$  Naive estimator | $\lambda$ | 0.939 | -0.061 | 0.043 | 0.051 |
|  | $\beta$ | 0.726 | -0.271 | 0.108 | 0.113 |
|  | $\beta_{m}$ | -0.267 | 0.133 | 0.031 | 0.034 |
|  | $\alpha$ | -1.157 | -0.357 | 0.194 | 0.190 |
|  | $\alpha_{m}$ | 0.139 | -0.261 | 0.051 | 0.052 |
|  | $\sigma_{b}$ | 0.420 | -0.080 | 0.081 | 0.129 |
| TMB with errors and response with misclassification  e ~ Normal (0, ${1.0}^{2}$)  $\left( \eta,\delta\right)=\left( 0.70,0.95 \right)$  Bayesian estimator | $\lambda$ | 1.036 | 0.036 | 0.093 | 0.098 |
|  | $\beta$ | 1.031 | 0.031 | 0.292 | 0.283 |
|  | $\beta_{m}$ | -0.517 | -0.117 | 0.161 | 0.123 |
|  | $\alpha$ | -0.791 | 0.009 | 0.591 | 0.384 |
|  | $\alpha_{m}$ | 0.438 | 0.038 | 0.321 | 0.179 |
|  | $\sigma_{b}$ | 0.451 | -0.049 | 0.143 | 0.230 |
|  | $\eta$ | 0.733 | 0.033 | 0.049 | 0.016 |
|  | $\delta$ | 0.964 | 0.014 | 0.021 | 0.003 |
| TMB with errors and response with misclassification  e ~ Normal (0, ${1.0}^{2}$)  $\left( \eta,\delta\right)=\left( 0.75,0.98 \right)$  Naive estimator | $\lambda$ | 0.949 | -0.051 | 0.067 | 0.081 |
|  | $\beta$ | 0.733 | -0.267 | 0.172 | 0.181 |
|  | $\beta_{m}$ | -0.268 | 0.132 | 0.049 | 0.052 |
|  | $\alpha$ | -1.125 | -0.325 | 0.311 | 0.258 |
|  | $\alpha_{m}$ | 0.166 | -0.234 | 0.084 | 0.079 |
|  | $\sigma_{b}$ | 0.414 | -0.086 | 0.116 | 0.192 |
| TMB with errors and response with misclassification  e ~ Normal (0, ${1.0}^{2}$)  $\left( \eta,\delta\right)=\left( 0.75,0.98 \right)$  Bayesian estimator | $\lambda$ | 1.045 | 0.045 | 0.094 | 0.104 |
|  | $\beta$ | 1.034 | 0.034 | 0.300 | 0.292 |
|  | $\beta_{m}$ | -0.520 | -0.120 | 0.160 | 0.121 |
|  | $\alpha$ | -0.734 | 0.066 | 0.607 | 0.386 |
|  | $\alpha_{m}$ | 0.470 | 0.070 | 0.333 | 0.191 |
|  | $\sigma_{b}$ | 0.474 | -0.026 | 0.158 | 0.230 |
|  | $\eta$ | 0.735 | -0.015 | 0.048 | 0.017 |
|  | $\delta$ | 0.964 | -0.016 | 0.021 | 0.003 |
| TMB with errors and response with misclassification  e ~ Normal (0, ${1.5}^{2}$)  $\left( \eta,\delta\right)=\left( 0.70,0.95 \right)$  Naive estimator | $\lambda$ | 0.922 | -0.078 | 0.043 | 0.051 |
|  | $\beta$ | 0.605 | -0.395 | 0.105 | 0.108 |
|  | $\beta_{m}$ | -0.193 | 0.207 | 0.026 | 0.027 |
|  | $\alpha$ | -1.133 | -0.333 | 0.188 | 0.173 |
|  | $\alpha_{m}$ | 0.110 | -0.290 | 0.044 | 0.044 |
|  | $\sigma_{b}$ | 0.432 | -0.068 | 0.083 | 0.124 |
| TMB with errors and response with misclassification  e ~ Normal (0, ${1.5}^{2}$)  $\left( \eta,\delta\right)=\left( 0.70,0.95 \right)$  Bayesian estimator | $\lambda$ | 1.035 | 0.035 | 0.095 | 0.111 |
|  | $\beta$ | 0.991 | -0.009 | 0.305 | 0.326 |
|  | $\beta_{m}$ | -0.468 | -0.068 | 0.160 | 0.146 |
|  | $\alpha$ | -0.755 | 0.045 | 0.623 | 0.389 |
|  | $\alpha_{m}$ | 0.402 | 0.002 | 0.338 | 0.193 |
|  | $\sigma_{b}$ | 0.455 | -0.045 | 0.148 | 0.257 |
|  | $\eta$ | 0.735 | 0.035 | 0.049 | 0.017 |
|  | $\delta$ | 0.964 | 0.014 | 0.021 | 0.003 |
| TMB with errors and response with misclassification  e ~ Normal (0, ${1.5}^{2}$)  $\left( \eta,\delta\right)=\left( 0.75,0.98 \right)$  Naive estimator | $\lambda$ | 0.927 | -0.073 | 0.067 | 0.078 |
|  | $\beta$ | 0.608 | -0.392 | 0.168 | 0.190 |
|  | $\beta_{m}$ | -0.194 | 0.206 | 0.041 | 0.042 |
|  | $\alpha$ | -1.082 | -0.282 | 0.303 | 0.268 |
|  | $\alpha_{m}$ | 0.130 | -0.270 | 0.072 | 0.064 |
|  | $\sigma_{b}$ | 0.417 | -0.083 | 0.122 | 0.183 |
| TMB with errors and response with misclassification  e ~ Normal (0, ${1.5}^{2}$)  $\left( \eta,\delta\right)=\left( 0.75,0.98 \right)$  Bayesian estimator | $\lambda$ | 1.025 | 0.025 | 0.097 | 0.116 |
|  | $\beta$ | 1.019 | 0.019 | 0.308 | 0.344 |
|  | $\beta_{m}$ | -0.468 | -0.068 | 0.160 | 0.144 |
|  | $\alpha$ | -0.762 | 0.038 | 0.641 | 0.383 |
|  | $\alpha_{m}$ | 0.442 | 0.042 | 0.345 | 0.199 |
|  | $\sigma_{b}$ | 0.455 | -0.045 | 0.154 | 0.263 |
|  | $\eta$ | 0.736 | -0.014 | 0.049 | 0.016 |
|  | $\delta$ | 0.963 | -0.017 | 0.022 | 0.003 |
| TMB with errors and response with misclassification  e ~ Normal (0, ${1.0}^{2}$)  $\left( \eta,\delta\right)=\left( 0.70,0.95 \right)$  Naive estimator | $\lambda$ | 0.939 | -0.061 | 0.043 | 0.052 |
|  | $\beta$ | 0.737 | -0.263 | 0.109 | 0.117 |
|  | $\beta_{m}$ | -0.265 | 0.135 | 0.031 | 0.034 |
|  | $\alpha$ | -1.169 | -0.369 | 0.194 | 0.187 |
|  | $\alpha_{m}$ | 0.148 | -0.252 | 0.052 | 0.052 |
|  | $\sigma_{b}$ | 0.421 | -0.079 | 0.082 | 0.126 |
| TMB with errors and response with misclassification  e ~ Normal (0, ${1.0}^{2}$)  $\left( \eta,\delta\right)=\left( 0.70,0.95 \right)$  Bayesian estimator | $\lambda$ | 1.044 | 0.044 | 0.093 | 0.110 |
|  | $\beta$ | 1.036 | 0.036 | 0.297 | 0.311 |
|  | $\beta_{m}$ | -0.524 | -0.124 | 0.158 | 0.122 |
|  | $\alpha$ | -0.758 | 0.042 | 0.586 | 0.379 |
|  | $\alpha_{m}$ | 0.406 | 0.006 | 0.302 | 0.185 |
|  | $\sigma_{b}$ | 0.455 | -0.045 | 0.144 | 0.248 |
|  | $\eta$ | 0.736 | 0.036 | 0.048 | 0.016 |
|  | $\delta$ | 0.964 | 0.014 | 0.021 | 0.003 |
| TMB with errors and response with misclassification  e ~ Normal (0, ${1.0}^{2}$)  $\left( \eta,\delta\right)=\left( 0.75,0.98 \right)$  Naive estimator | $\lambda$ | 0.957 | -0.043 | 0.070 | 0.084 |
|  | $\beta$ | 0.746 | -0.254 | 0.175 | 0.192 |
|  | $\beta_{m}$ | -0.274 | 0.126 | 0.050 | 0.058 |
|  | $\alpha$ | -1.113 | -0.313 | 0.311 | 0.261 |
|  | $\alpha_{m}$ | 0.170 | -0.230 | 0.085 | 0.080 |
|  | $\sigma_{b}$ | 0.428 | -0.072 | 0.119 | 0.198 |
| TMB with errors and response with misclassification  e ~ Normal (0, ${1.0}^{2}$)  $\left( \eta,\delta\right)=\left( 0.75,0.98 \right)$  Bayesian estimator | $\lambda$ | 1.049 | 0.049 | 0.094 | 0.117 |
|  | $\beta$ | 1.044 | 0.044 | 0.296 | 0.279 |
|  | $\beta_{m}$ | -0.511 | -0.111 | 0.152 | 0.129 |
|  | $\alpha$ | -0.812 | -0.012 | 0.602 | 0.395 |
|  | $\alpha_{m}$ | 0.471 | 0.071 | 0.313 | 0.175 |
|  | $\sigma_{b}$ | 0.462 | -0.038 | 0.149 | 0.261 |
|  | $\eta$ | 0.737 | -0.013 | 0.048 | 0.017 |
|  | $\delta$ | 0.964 | -0.016 | 0.021 | 0.004 |
| TMB with errors and response with misclassification  e ~ Laplace (0, ${1.5}^{2}$)  $\left( \eta,\delta\right)=\left( 0.70,0.95 \right)$  Naive estimator | $\lambda$ | 0.919 | -0.081 | 0.043 | 0.050 |
|  | $\beta$ | 0.601 | -0.399 | 0.106 | 0.120 |
|  | $\beta_{m}$ | -0.193 | 0.207 | 0.026 | 0.030 |
|  | $\alpha$ | -1.130 | -0.330 | 0.190 | 0.180 |
|  | $\alpha_{m}$ | 0.111 | -0.289 | 0.044 | 0.045 |
|  | $\sigma_{b}$ | 0.430 | -0.070 | 0.084 | 0.126 |
| TMB with errors and response with misclassification  e ~ Laplace (0, ${1.5}^{2}$)  $\left( \eta,\delta\right)=\left( 0.70,0.95 \right)$  Bayesian estimator | $\lambda$ | 1.016 | 0.016 | 0.090 | 0.106 |
|  | $\beta$ | 0.970 | -0.030 | 0.292 | 0.319 |
|  | $\beta_{m}$ | -0.440 | -0.040 | 0.145 | 0.146 |
|  | $\alpha$ | -0.802 | -0.002 | 0.622 | 0.396 |
|  | $\alpha_{m}$ | 0.379 | -0.021 | 0.324 | 0.188 |
|  | $\sigma_{b}$ | 0.446 | -0.054 | 0.143 | 0.244 |
|  | $\eta$ | 0.734 | 0.034 | 0.049 | 0.016 |
|  | $\delta$ | 0.964 | 0.014 | 0.021 | 0.004 |
| TMB with errors and response with misclassification  e ~ Laplace (0, ${1.5}^{2}$)  $\left( \eta,\delta\right)=\left( 0.75,0.98 \right)$  Naive estimator | $\lambda$ | 0.928 | -0.072 | 0.067 | 0.082 |
|  | $\beta$ | 0.617 | -0.383 | 0.167 | 0.190 |
|  | $\beta_{m}$ | -0.196 | 0.204 | 0.041 | 0.048 |
|  | $\alpha$ | -1.074 | -0.274 | 0.304 | 0.284 |
|  | $\alpha_{m}$ | 0.128 | -0.272 | 0.073 | 0.062 |
|  | $\sigma_{b}$ | 0.416 | -0.084 | 0.119 | 0.187 |
| TMB with errors and response with misclassification  e ~ Laplace (0, ${1.5}^{2}$)  $\left( \eta,\delta\right)=\left( 0.75,0.98 \right)$  Bayesian estimator | $\lambda$ | 1.031 | 0.031 | 0.096 | 0.116 |
|  | $\beta$ | 0.991 | -0.009 | 0.293 | 0.300 |
|  | $\beta_{m}$ | -0.451 | -0.051 | 0.155 | 0.154 |
|  | $\alpha$ | -0.762 | 0.038 | 0.619 | 0.415 |
|  | $\alpha_{m}$ | 0.430 | 0.030 | 0.329 | 0.185 |
|  | $\sigma_{b}$ | 0.453 | -0.047 | 0.149 | 0.258 |
|  | $\eta$ | 0.735 | -0.015 | 0.049 | 0.016 |
|  | $\delta$ | 0.963 | -0.017 | 0.021 | 0.003 |
| TMB with errors and response with misclassification  e ~ Extreme (0, ${1.0}^{2}$)  $\left( \eta,\delta\right)=\left( 0.70,0.95 \right)$  Naive estimator | $\lambda$ | 0.957 | -0.043 | 0.045 | 0.056 |
|  | $\beta$ | 0.664 | -0.336 | 0.113 | 0.131 |
|  | $\beta_{m}$ | -0.207 | 0.193 | 0.036 | 0.041 |
|  | $\alpha$ | -1.098 | -0.298 | 0.202 | 0.190 |
|  | $\alpha_{m}$ | 0.091 | -0.309 | 0.064 | 0.053 |
|  | $\sigma_{b}$ | 0.450 | -0.050 | 0.084 | 0.129 |
| TMB with errors and response with misclassification  e ~ Extreme (0, ${1.0}^{2}$)  $\left( \eta,\delta\right)=\left( 0.70,0.95 \right)$  Bayesian estimator | $\lambda$ | 1.046 | 0.046 | 0.096 | 0.115 |
|  | $\beta$ | 1.045 | 0.045 | 0.297 | 0.319 |
|  | $\beta_{m}$ | -0.527 | -0.127 | 0.165 | 0.136 |
|  | $\alpha$ | -0.785 | 0.015 | 0.599 | 0.391 |
|  | $\alpha_{m}$ | 0.436 | 0.036 | 0.318 | 0.196 |
|  | $\sigma_{b}$ | 0.445 | -0.055 | 0.144 | 0.258 |
|  | $\eta$ | 0.736 | 0.036 | 0.048 | 0.016 |
|  | $\delta$ | 0.964 | 0.014 | 0.021 | 0.003 |
| TMB with errors and response with misclassification  e ~ Extreme (0, ${1.0}^{2}$)  $\left( \eta,\delta\right)=\left( 0.75,0.98 \right)$  Naive estimator | $\lambda$ | 0.949 | -0.051 | 0.069 | 0.082 |
|  | $\beta$ | 0.749 | -0.251 | 0.174 | 0.202 |
|  | $\beta_{m}$ | -0.263 | 0.137 | 0.049 | 0.053 |
|  | $\alpha$ | -1.128 | -0.328 | 0.312 | 0.265 |
|  | $\alpha_{m}$ | 0.173 | -0.227 | 0.085 | 0.079 |
|  | $\sigma_{b}$ | 0.419 | -0.081 | 0.120 | 0.190 |
| TMB with errors and response with misclassification  e ~ Extreme (0, ${1.0}^{2}$)  $\left( \eta,\delta\right)=\left( 0.75,0.98 \right)$  Bayesian estimator | $\lambda$ | 1.053 | 0.053 | 0.097 | 0.109 |
|  | $\beta$ | 1.060 | 0.060 | 0.293 | 0.290 |
|  | $\beta_{m}$ | -0.517 | -0.117 | 0.157 | 0.132 |
|  | $\alpha$ | -0.820 | -0.020 | 0.616 | 0.390 |
|  | $\alpha_{m}$ | 0.473 | 0.073 | 0.320 | 0.179 |
|  | $\sigma_{b}$ | 0.486 | -0.014 | 0.158 | 0.253 |
|  | $\eta$ | 0.738 | -0.012 | 0.048 | 0.016 |
|  | $\delta$ | 0.964 | -0.016 | 0.021 | 0.004 |
| TMB with errors and response with misclassification  e ~ Extreme (0, ${1.5}^{2}$)  $\left( \eta,\delta\right)=\left( 0.70,0.95 \right)$  Naive estimator | $\lambda$ | 0.920 | -0.080 | 0.044 | 0.051 |
|  | $\beta$ | 0.588 | -0.412 | 0.106 | 0.117 |
|  | $\beta_{m}$ | -0.183 | 0.217 | 0.025 | 0.028 |
|  | $\alpha$ | -1.133 | -0.333 | 0.192 | 0.195 |
|  | $\alpha_{m}$ | 0.111 | -0.289 | 0.045 | 0.046 |
|  | $\sigma_{b}$ | 0.446 | -0.054 | 0.085 | 0.122 |
| TMB with errors and response with misclassification  e ~ Extreme (0, ${1.5}^{2}$)  $\left( \eta,\delta\right)=\left( 0.70,0.95 \right)$  Bayesian estimator | $\lambda$ | 1.038 | 0.038 | 0.095 | 0.111 |
|  | $\beta$ | 0.998 | -0.002 | 0.297 | 0.337 |
|  | $\beta_{m}$ | -0.469 | -0.069 | 0.158 | 0.156 |
|  | $\alpha$ | -0.774 | 0.026 | 0.614 | 0.371 |
|  | $\alpha_{m}$ | 0.410 | 0.010 | 0.337 | 0.193 |
|  | $\sigma_{b}$ | 0.476 | -0.024 | 0.151 | 0.243 |
|  | $\eta$ | 0.734 | 0.034 | 0.049 | 0.016 |
|  | $\delta$ | 0.964 | 0.014 | 0.021 | 0.003 |
| TMB with errors and response with misclassification  e ~ Extreme (0, ${1.5}^{2}$)  $\left( \eta,\delta\right)=\left( 0.75,0.98 \right)$  Naive estimator | $\lambda$ | 0.928 | -0.072 | 0.068 | 0.083 |
|  | $\beta$ | 0.577 | -0.423 | 0.169 | 0.192 |
|  | $\beta_{m}$ | -0.183 | 0.217 | 0.040 | 0.047 |
|  | $\alpha$ | -1.102 | -0.302 | 0.306 | 0.266 |
|  | $\alpha_{m}$ | 0.133 | -0.267 | 0.073 | 0.068 |
|  | $\sigma_{b}$ | 0.436 | -0.064 | 0.123 | 0.196 |
| TMB with errors and response with misclassification  e ~ Extreme (0, ${1.5}^{2}$)  $\left( \eta,\delta\right)=\left( 0.75,0.98 \right)$  Bayesian estimator | $\lambda$ | 1.029 | 0.029 | 0.095 | 0.099 |
|  | $\beta$ | 0.959 | -0.041 | 0.295 | 0.329 |
|  | $\beta_{m}$ | -0.444 | -0.044 | 0.156 | 0.148 |
|  | $\alpha$ | -0.814 | -0.014 | 0.638 | 0.407 |
|  | $\alpha_{m}$ | 0.439 | 0.039 | 0.339 | 0.181 |
|  | $\sigma_{b}$ | 0.475 | -0.025 | 0.155 | 0.241 |
|  | $\eta$ | 0.737 | -0.013 | 0.048 | 0.016 |
|  | $\delta$ | 0.963 | -0.017 | 0.022 | 0.004 |
| TMB ~ Laplace (mean = 1, var = ${1.5}^{2}$) | | | | | |
| True-data  Bayesian estimator | $\lambda$ | 1.015 | 0.015 | 0.076 | 0.099 |
|  | $\beta$ | 1.011 | 0.011 | 0.193 | 0.215 |
|  | $\beta_{m}$ | -0.404 | -0.004 | 0.065 | 0.074 |
|  | $\alpha$ | -0.853 | -0.053 | 0.328 | 0.304 |
|  | $\alpha_{m}$ | 0.426 | 0.026 | 0.116 | 0.116 |
|  | $\sigma_{b}$ | 0.476 | -0.024 | 0.135 | 0.208 |
| TMB with errors  e ~ Normal (0, ${1.0}^{2}$)  Naive estimator | $\lambda$ | 0.963 | -0.037 | 0.070 | 0.082 |
|  | $\beta$ | 0.760 | -0.240 | 0.177 | 0.199 |
|  | $\beta_{m}$ | -0.270 | 0.130 | 0.051 | 0.054 |
|  | $\alpha$ | -0.637 | 0.163 | 0.303 | 0.292 |
|  | $\alpha_{m}$ | 0.286 | -0.114 | 0.089 | 0.089 |
|  | $\sigma_{b}$ | 0.447 | -0.053 | 0.123 | 0.204 |
| TMB with errors  e ~ Normal (0, ${1.0}^{2}$)  Bayesian estimator | $\lambda$ | 1.030 | 0.030 | 0.089 | 0.101 |
|  | $\beta$ | 1.021 | 0.021 | 0.272 | 0.291 |
|  | $\beta_{m}$ | -0.467 | -0.067 | 0.133 | 0.124 |
|  | $\alpha$ | -0.783 | 0.017 | 0.388 | 0.331 |
|  | $\alpha_{m}$ | 0.443 | 0.043 | 0.186 | 0.159 |
|  | $\sigma_{b}$ | 0.462 | -0.038 | 0.140 | 0.223 |
| TMB with errors  e ~ Normal (0, ${1.0}^{2}$)  Corrected score estimator | $\lambda$ | 1.001 | 0.001 | 0.056 | 0.092 |
|  | $\beta$ | 0.945 | -0.055 | 0.152 | 0.233 |
|  | $\beta_{m}$ | -0.385 | 0.015 | 0.054 | 0.093 |
|  | $\alpha$ | -0.783 | 0.017 | 0.316 | 0.349 |
|  | $\alpha_{m}$ | 0.384 | -0.016 | 0.108 | 0.144 |
|  | $\sigma_{b}$ | 0.479 | -0.021 | 0.044 | 0.207 |
| TMB with errors  e ~ Normal (0, ${1.5}^{2}$)  Naive estimator | $\lambda$ | 0.931 | -0.069 | 0.067 | 0.078 |
|  | $\beta$ | 0.605 | -0.395 | 0.168 | 0.174 |
|  | $\beta_{m}$ | -0.192 | 0.208 | 0.042 | 0.044 |
|  | $\alpha$ | -0.518 | 0.282 | 0.291 | 0.273 |
|  | $\alpha_{m}$ | 0.204 | -0.196 | 0.073 | 0.072 |
|  | $\sigma_{b}$ | 0.415 | -0.085 | 0.119 | 0.188 |
| TMB with errors  e ~ Normal (0, ${1.5}^{2}$)  Bayesian estimator | $\lambda$ | 1.023 | 0.023 | 0.091 | 0.098 |
|  | $\beta$ | 0.985 | -0.015 | 0.292 | 0.303 |
|  | $\beta_{m}$ | -0.445 | -0.045 | 0.150 | 0.141 |
|  | $\alpha$ | -0.761 | 0.039 | 0.410 | 0.342 |
|  | $\alpha_{m}$ | 0.425 | 0.025 | 0.212 | 0.163 |
|  | $\sigma_{b}$ | 0.458 | -0.042 | 0.138 | 0.234 |
| TMB with errors  e ~ Normal (0, ${1.5}^{2}$)  Corrected score estimator | $\lambda$ | 0.955 | -0.045 | 0.055 | 0.093 |
|  | $\beta$ | 0.810 | -0.190 | 0.162 | 0.326 |
|  | $\beta_{m}$ | -0.300 | 0.100 | 0.055 | 0.162 |
|  | $\alpha$ | -0.808 | -0.008 | 0.318 | 0.388 |
|  | $\alpha_{m}$ | 0.419 | 0.019 | 0.112 | 0.166 |
|  | $\sigma_{b}$ | 0.408 | -0.092 | 0.042 | 0.196 |
| TMB with errors  e ~ Laplace (0, ${1.0}^{2}$)  Naive estimator | $\lambda$ | 0.954 | -0.046 | 0.070 | 0.083 |
|  | $\beta$ | 0.755 | -0.245 | 0.176 | 0.194 |
|  | $\beta_{m}$ | -0.272 | 0.128 | 0.051 | 0.054 |
|  | $\alpha$ | -0.624 | 0.176 | 0.301 | 0.288 |
|  | $\alpha_{m}$ | 0.280 | -0.120 | 0.089 | 0.092 |
|  | $\sigma_{b}$ | 0.436 | -0.064 | 0.123 | 0.190 |
| TMB with errors  e ~ Laplace (0, ${1.0}^{2}$)  Bayesian estimator | $\lambda$ | 1.026 | 0.026 | 0.087 | 0.104 |
|  | $\beta$ | 1.014 | 0.014 | 0.263 | 0.281 |
|  | $\beta_{m}$ | -0.453 | -0.053 | 0.124 | 0.134 |
|  | $\alpha$ | -0.799 | 0.001 | 0.384 | 0.352 |
|  | $\alpha_{m}$ | 0.445 | 0.045 | 0.178 | 0.148 |
|  | $\sigma_{b}$ | 0.456 | -0.044 | 0.137 | 0.229 |
| TMB with errors  e ~ Laplace (0, ${1.0}^{2}$)  Corrected score estimator | $\lambda$ | 1.005 | 0.005 | 0.056 | 0.092 |
|  | $\beta$ | 0.926 | -0.074 | 0.151 | 0.233 |
|  | $\beta_{m}$ | -0.381 | 0.019 | 0.052 | 0.105 |
|  | $\alpha$ | -0.816 | -0.016 | 0.325 | 0.374 |
|  | $\alpha_{m}$ | 0.392 | -0.008 | 0.114 | 0.172 |
|  | $\sigma_{b}$ | 0.501 | 0.001 | 0.044 | 0.196 |
| TMB with errors  e ~ Laplace (0, ${1.5}^{2}$)  Naive estimator | $\lambda$ | 0.935 | -0.065 | 0.069 | 0.084 |
|  | $\beta$ | 0.609 | -0.391 | 0.170 | 0.182 |
|  | $\beta_{m}$ | -0.194 | 0.206 | 0.041 | 0.046 |
|  | $\alpha$ | -0.525 | 0.275 | 0.292 | 0.289 |
|  | $\alpha_{m}$ | 0.213 | -0.187 | 0.075 | 0.081 |
|  | $\sigma_{b}$ | 0.429 | -0.071 | 0.125 | 0.194 |
| TMB with errors  e ~ Laplace (0, ${1.5}^{2}$)  Bayesian estimator | $\lambda$ | 1.005 | 0.005 | 0.086 | 0.105 |
|  | $\beta$ | 0.939 | -0.061 | 0.270 | 0.307 |
|  | $\beta_{m}$ | -0.401 | -0.001 | 0.128 | 0.152 |
|  | $\alpha$ | -0.703 | 0.097 | 0.384 | 0.337 |
|  | $\alpha_{m}$ | 0.374 | -0.026 | 0.182 | 0.171 |
|  | $\sigma_{b}$ | 0.454 | -0.046 | 0.138 | 0.217 |
| TMB with errors  e ~ Laplace (0, ${1.5}^{2}$)  Corrected score estimator | $\lambda$ | 0.972 | -0.028 | 0.055 | 0.106 |
|  | $\beta$ | 0.794 | -0.206 | 0.152 | 0.306 |
|  | $\beta_{m}$ | -0.294 | 0.106 | 0.046 | 0.151 |
|  | $\alpha$ | -0.805 | -0.005 | 0.343 | 0.434 |
|  | $\alpha_{m}$ | 0.416 | 0.016 | 0.137 | 0.201 |
|  | $\sigma_{b}$ | 0.476 | -0.024 | 0.043 | 0.191 |
| TMB with errors  e ~ Extreme (0, ${1.0}^{2}$)  Naive estimator | $\lambda$ | 0.963 | -0.037 | 0.070 | 0.086 |
|  | $\beta$ | 0.737 | -0.263 | 0.177 | 0.195 |
|  | $\beta_{m}$ | -0.264 | 0.136 | 0.050 | 0.057 |
|  | $\alpha$ | -0.653 | 0.147 | 0.306 | 0.303 |
|  | $\alpha_{m}$ | 0.293 | -0.107 | 0.090 | 0.093 |
|  | $\sigma_{b}$ | 0.452 | -0.048 | 0.125 | 0.198 |
| TMB with errors  e ~ Extreme (0, ${1.0}^{2}$)  Bayesian estimator | $\lambda$ | 1.022 | 0.022 | 0.087 | 0.099 |
|  | $\beta$ | 0.992 | -0.008 | 0.265 | 0.271 |
|  | $\beta_{m}$ | -0.449 | -0.049 | 0.127 | 0.128 |
|  | $\alpha$ | -0.792 | 0.008 | 0.384 | 0.341 |
|  | $\alpha_{m}$ | 0.447 | 0.047 | 0.183 | 0.149 |
|  | $\sigma_{b}$ | 0.460 | -0.040 | 0.136 | 0.220 |
| TMB with errors  e ~ Extreme (0, ${1.0}^{2}$)  Corrected score estimator | $\lambda$ | 1.017 | 0.017 | 0.056 | 0.102 |
|  | $\beta$ | 0.933 | -0.067 | 0.149 | 0.268 |
|  | $\beta_{m}$ | -0.369 | 0.031 | 0.049 | 0.103 |
|  | $\alpha$ | -0.796 | 0.004 | 0.319 | 0.381 |
|  | $\alpha_{m}$ | 0.387 | -0.013 | 0.112 | 0.161 |
|  | $\sigma_{b}$ | 0.528 | 0.028 | 0.045 | 0.203 |
| TMB with errors  e ~ Extreme (0, ${1.5}^{2}$)  Naive estimator | $\lambda$ | 0.930 | -0.070 | 0.067 | 0.082 |
|  | $\beta$ | 0.578 | -0.422 | 0.167 | 0.190 |
|  | $\beta_{m}$ | -0.182 | 0.218 | 0.040 | 0.046 |
|  | $\alpha$ | -0.557 | 0.243 | 0.293 | 0.277 |
|  | $\alpha_{m}$ | 0.205 | -0.195 | 0.074 | 0.070 |
|  | $\sigma_{b}$ | 0.423 | -0.077 | 0.120 | 0.189 |
| TMB with errors  e ~ Extreme (0, ${1.5}^{2}$)  Bayesian estimator | $\lambda$ | 1.011 | 0.011 | 0.089 | 0.104 |
|  | $\beta$ | 0.957 | -0.043 | 0.280 | 0.303 |
|  | $\beta_{m}$ | -0.430 | 0.030 | 0.142 | 0.160 |
|  | $\alpha$ | -0.740 | 0.060 | 0.402 | 0.355 |
|  | $\alpha_{m}$ | 0.408 | 0.008 | 0.202 | 0.167 |
|  | $\sigma_{b}$ | 0.445 | -0.055 | 0.136 | 0.226 |
| TMB with errors  e ~ Extreme (0, ${1.5}^{2}$)  Corrected score estimator | $\lambda$ | 0.963 | -0.037 | 0.054 | 0.098 |
|  | $\beta$ | 0.746 | -0.254 | 0.143 | 0.272 |
|  | $\beta_{m}$ | -0.269 | 0.131 | 0.039 | 0.119 |
|  | $\alpha$ | -0.782 | 0.018 | 0.337 | 0.401 |
|  | $\alpha_{m}$ | 0.395 | -0.005 | 0.111 | 0.193 |
|  | $\sigma_{b}$ | 0.470 | -0.030 | 0.044 | 0.206 |
| Response with misclassification  $\left( \eta,\delta\right)=\left( 0.70,0.95 \right)$  Naive estimator | $\lambda$ | 0.984 | -0.016 | 0.046 | 0.054 |
|  | $\beta$ | 0.967 | -0.033 | 0.116 | 0.129 |
|  | $\beta_{m}$ | -0.394 | 0.006 | 0.039 | 0.040 |
|  | $\alpha$ | -1.240 | -0.440 | 0.204 | 0.174 |
|  | $\alpha_{m}$ | 0.196 | -0.204 | 0.063 | 0.058 |
|  | $\sigma_{b}$ | 0.426 | -0.074 | 0.085 | 0.133 |
| Response with misclassification  $\left( \eta,\delta\right)=\left( 0.70,0.95 \right)$  Bayesian estimator | $\lambda$ | 1.009 | 0.009 | 0.051 | 0.069 |
|  | $\beta$ | 1.007 | 0.007 | 0.124 | 0.143 |
|  | $\beta_{m}$ | -0.401 | -0.001 | 0.041 | 0.044 |
|  | $\alpha$ | -0.834 | -0.034 | 0.351 | 0.328 |
|  | $\alpha_{m}$ | 0.402 | 0.002 | 0.125 | 0.122 |
|  | $\sigma_{b}$ | 0.488 | -0.012 | 0.097 | 0.157 |
|  | $\eta$ | 0.729 | 0.029 | 0.044 | 0.024 |
|  | $\delta$ | 0.964 | 0.014 | 0.020 | 0.005 |
| Response with misclassification  $\left( \eta,\delta\right)=\left( 0.75,0.98 \right)$  Naive estimator | $\lambda$ | 0.985 | -0.015 | 0.045 | 0.054 |
|  | $\beta$ | 0.973 | -0.027 | 0.115 | 0.123 |
|  | $\beta_{m}$ | -0.395 | 0.005 | 0.039 | 0.042 |
|  | $\alpha$ | -1.250 | -0.450 | 0.205 | 0.188 |
|  | $\alpha_{m}$ | 0.229 | -0.171 | 0.064 | 0.063 |
|  | $\sigma_{b}$ | 0.429 | -0.071 | 0.083 | 0.125 |
| Response with misclassification  $\left( \eta,\delta\right)=\left( 0.75,0.98 \right)$  Bayesian estimator | $\lambda$ | 1.015 | 0.015 | 0.052 | 0.066 |
|  | $\beta$ | 1.015 | 0.015 | 0.126 | 0.146 |
|  | $\beta_{m}$ | -0.406 | -0.006 | 0.042 | 0.047 |
|  | $\alpha$ | -0.877 | -0.077 | 0.357 | 0.342 |
|  | $\alpha_{m}$ | 0.464 | 0.064 | 0.135 | 0.128 |
|  | $\sigma_{b}$ | 0.501 | 0.001 | 0.101 | 0.160 |
|  | $\eta$ | 0.733 | -0.017 | 0.044 | 0.024 |
|  | $\delta$ | 0.964 | -0.016 | 0.021 | 0.006 |
| TMB with errors and response with misclassification  e ~ Normal (0, ${1.0}^{2}$)  $\left( \eta,\delta\right)=\left( 0.70,0.95 \right)$  Naive estimator | $\lambda$ | 0.943 | -0.057 | 0.043 | 0.054 |
|  | $\beta$ | 0.734 | -0.266 | 0.108 | 0.120 |
|  | $\beta_{m}$ | -0.268 | 0.132 | 0.031 | 0.034 |
|  | $\alpha$ | -1.178 | -0.378 | 0.196 | 0.192 |
|  | $\alpha_{m}$ | 0.139 | -0.261 | 0.053 | 0.047 |
|  | $\sigma_{b}$ | 0.423 | -0.077 | 0.081 | 0.134 |
| TMB with errors and response with misclassification  e ~ Normal (0, ${1.0}^{2}$)  $\left( \eta,\delta\right)=\left( 0.70,0.95 \right)$  Bayesian estimator | $\lambda$ | 1.041 | 0.041 | 0.092 | 0117 |
|  | $\beta$ | 1.043 | 0.043 | 0.276 | 0.307 |
|  | $\beta_{m}$ | -0.477 | -0.077 | 0.137 | 0.124 |
|  | $\alpha$ | -0.790 | 0.010 | 0.588 | 0.391 |
|  | $\alpha_{m}$ | 0.416 | 0.016 | 0.294 | 0.179 |
|  | $\sigma_{b}$ | 0.472 | -0.028 | 0.150 | 0.262 |
|  | $\eta$ | 0.733 | 0.033 | 0.049 | 0.017 |
|  | $\delta$ | 0.964 | 0.014 | 0.021 | 0.004 |
| TMB with errors and response with misclassification  e ~ Normal (0, ${1.0}^{2}$)  $\left( \eta,\delta\right)=\left( 0.75,0.98 \right)$  Naive estimator | $\lambda$ | 0.949 | -0.051 | 0.068 | 0.081 |
|  | $\beta$ | 0.721 | -0.279 | 0.172 | 0.181 |
|  | $\beta_{m}$ | -0.266 | 0.134 | 0.050 | 0.052 |
|  | $\alpha$ | -1.127 | -0.327 | 0.312 | 0.262 |
|  | $\alpha_{m}$ | 0.158 | -0.242 | 0.086 | 0.076 |
|  | $\sigma_{b}$ | 0.407 | -0.093 | 0.119 | 0.189 |
| TMB with errors and response with misclassification  e ~ Normal (0, ${1.0}^{2}$)  $\left( \eta,\delta\right)=\left( 0.75,0.98 \right)$  Bayesian estimator | $\lambda$ | 1.035 | 0.035 | 0.091 | 0.112 |
|  | $\beta$ | 1.025 | 0.025 | 0.277 | 0.287 |
|  | $\beta_{m}$ | -0.465 | -0.065 | 0.131 | 0.126 |
|  | $\alpha$ | -0.802 | -0.002 | 0.599 | 0.389 |
|  | $\alpha_{m}$ | 0.449 | 0.049 | 0.297 | 0.180 |
|  | $\sigma_{b}$ | 0.483 | -0.017 | 0.155 | 0.244 |
|  | $\eta$ | 0.735 | -0.015 | 0.048 | 0.017 |
|  | $\delta$ | 0.964 | -0.016 | 0.021 | 0.003 |
| TMB with errors and response with misclassification  e ~ Normal (0, ${1.5}^{2}$)  $\left( \eta,\delta\right)=\left( 0.70,0.95 \right)$  Naive estimator | $\lambda$ | 0.926 | -0.074 | 0.043 | 0.050 |
|  | $\beta$ | 0.598 | -0.402 | 0.105 | 0.109 |
|  | $\beta_{m}$ | -0.190 | 0.210 | 0.026 | 0.027 |
|  | $\alpha$ | -1.112 | -0.312 | 0.190 | 0.190 |
|  | $\alpha_{m}$ | 0.103 | -0.297 | 0.045 | 0.043 |
|  | $\sigma_{b}$ | 0.436 | -0.064 | 0.082 | 0.121 |
| TMB with errors and response with misclassification  e ~ Normal (0, ${1.5}^{2}$)  $\left( \eta,\delta\right)=\left( 0.70,0.95 \right)$  Bayesian estimator | $\lambda$ | 1.029 | 0.029 | 0.094 | 0.115 |
|  | $\beta$ | 0.967 | -0.033 | 0.294 | 0.322 |
|  | $\beta_{m}$ | -0.438 | -0.038 | 0.148 | 0.135 |
|  | $\alpha$ | -0.782 | 0.018 | 0.633 | 0.398 |
|  | $\alpha_{m}$ | 0.405 | 0.005 | 0.337 | 0.193 |
|  | $\sigma_{b}$ | 0.461 | -0.039 | 0.149 | 0.255 |
|  | $\eta$ | 0.734 | 0.034 | 0.049 | 0.017 |
|  | $\delta$ | 0.964 | 0.014 | 0.021 | 0.003 |
| TMB with errors and response with misclassification  e ~ Normal (0, ${1.5}^{2}$)  $\left( \eta,\delta\right)=\left( 0.75,0.98 \right)$  Naive estimator | $\lambda$ | 0.933 | -0.067 | 0.067 | 0.080 |
|  | $\beta$ | 0.605 | -0.395 | 0.168 | 0.183 |
|  | $\beta_{m}$ | -0.194 | 0.206 | 0.041 | 0.043 |
|  | $\alpha$ | -1.067 | -0.267 | 0.303 | 0.253 |
|  | $\alpha_{m}$ | 0.125 | -0.275 | 0.073 | 0.066 |
|  | $\sigma_{b}$ | 0.416 | -0.084 | 0.119 | 0.190 |
| TMB with errors and response with misclassification  e ~ Normal (0, ${1.5}^{2}$)  $\left( \eta,\delta\right)=\left( 0.75,0.98 \right)$  Bayesian estimator | $\lambda$ | 1.033 | 0.033 | 0.096 | 0.113 |
|  | $\beta$ | 1.047 | 0.047 | 0.304 | 0.344 |
|  | $\beta_{m}$ | -0.460 | -0.060 | 0.156 | 0.144 |
|  | $\alpha$ | -0.757 | 0.043 | 0.634 | 0.409 |
|  | $\alpha_{m}$ | 0.414 | 0.014 | 0.326 | 0.196 |
|  | $\sigma_{b}$ | 0.481 | -0.019 | 0.152 | 0.257 |
|  | $\eta$ | 0.735 | -0.015 | 0.048 | 0.016 |
|  | $\delta$ | 0.964 | -0.016 | 0.021 | 0.003 |
| TMB with errors and response with misclassification  e ~ Laplace (0, ${1.0}^{2}$)  $\left( \eta,\delta\right)=\left( 0.70,0.95 \right)$  Naive estimator | $\lambda$ | 0.942 | -0.058 | 0.044 | 0.056 |
|  | $\beta$ | 0.732 | -0.268 | 0.109 | 0.119 |
|  | $\beta_{m}$ | -0.268 | 0.132 | 0.031 | 0.033 |
|  | $\alpha$ | -1.153 | -0.353 | 0.195 | 0.192 |
|  | $\alpha_{m}$ | 0.138 | -0.262 | 0.053 | 0.054 |
|  | $\sigma_{b}$ | 0.424 | -0.076 | 0.083 | 0.137 |
| TMB with errors and response with misclassification  e ~ Laplace (0, ${1.0}^{2}$)  $\left( \eta,\delta\right)=\left( 0.70,0.95 \right)$  Bayesian estimator | $\lambda$ | 1.026 | 0.026 | 0.088 | 0.112 |
|  | $\beta$ | 1.012 | 0.012 | 0.265 | 0.308 |
|  | $\beta_{m}$ | -0.451 | -0.051 | 0.124 | 0.136 |
|  | $\alpha$ | -0.801 | -0.001 | 0.594 | 0.382 |
|  | $\alpha_{m}$ | 0.407 | 0.007 | 0.285 | 0.183 |
|  | $\sigma_{b}$ | 0.459 | -0.041 | 0.144 | 0.247 |
|  | $\eta$ | 0.732 | 0.032 | 0.048 | 0.018 |
|  | $\delta$ | 0.964 | 0.014 | 0.021 | 0.004 |
| TMB with errors and response with misclassification  e ~ Laplace (0, ${1.0}^{2}$)  $\left( \eta,\delta\right)=\left( 0.75,0.98 \right)$  Naive estimator | $\lambda$ | 0.955 | -0.045 | 0.069 | 0.083 |
|  | $\beta$ | 0.740 | -0.260 | 0.174 | 0.194 |
|  | $\beta_{m}$ | -0.268 | 0.132 | 0.050 | 0.056 |
|  | $\alpha$ | -1.139 | -0.339 | 0.313 | 0.266 |
|  | $\alpha_{m}$ | 0.170 | -0.230 | 0.086 | 0.076 |
|  | $\sigma_{b}$ | 0.424 | -0.076 | 0.120 | 0.193 |
| TMB with errors and response with misclassification  e ~ Laplace (0, ${1.0}^{2}$)  $\left( \eta,\delta\right)=\left( 0.75,0.98 \right)$  Bayesian estimator | $\lambda$ | 1.040 | 0.040 | 0.093 | 0.117 |
|  | $\beta$ | 1.028 | 0.028 | 0.269 | 0.286 |
|  | $\beta_{m}$ | -0.448 | -0.048 | 0.123 | 0.129 |
|  | $\alpha$ | -0.775 | 0.025 | 0.608 | 0.397 |
|  | $\alpha_{m}$ | 0.453 | 0.053 | 0.304 | 0.187 |
|  | $\sigma_{b}$ | 0.501 | 0.001 | 0.157 | 0.267 |
|  | $\eta$ | 0.734 | -0.016 | 0.048 | 0.017 |
|  | $\delta$ | 0.964 | -0.016 | 0.021 | 0.004 |
| TMB with errors and response with misclassification  e ~ Laplace (0, ${1.5}^{2}$)  $\left( \eta,\delta\right)=\left( 0.70,0.95 \right)$  Naive estimator | $\lambda$ | 0.921 | -0.079 | 0.043 | 0.055 |
|  | $\beta$ | 0.598 | -0.402 | 0.105 | 0.114 |
|  | $\beta_{m}$ | -0.190 | 0.210 | 0.026 | 0.030 |
|  | $\alpha$ | -1.106 | -0.306 | 0.189 | 0.179 |
|  | $\alpha_{m}$ | 0.096 | -0.304 | 0.045 | 0.043 |
|  | $\sigma_{b}$ | 0.425 | -0.075 | 0.084 | 0.133 |
| TMB with errors and response with misclassification  e ~ Laplace (0, ${1.5}^{2}$)  $\left( \eta,\delta\right)=\left( 0.70,0.95 \right)$  Bayesian estimator | $\lambda$ | 1.019 | 0.019 | 0.091 | 0.114 |
|  | $\beta$ | 0.923 | -0.077 | 0.271 | 0.291 |
|  | $\beta_{m}$ | -0.405 | -0.005 | 0.132 | 0.158 |
|  | $\alpha$ | -0.747 | 0.053 | 0.608 | 0.387 |
|  | $\alpha_{m}$ | 0.375 | -0.025 | 0.302 | 0.195 |
|  | $\sigma_{b}$ | 0.476 | -0.024 | 0.150 | 0.250 |
|  | $\eta$ | 0.734 | 0.034 | 0.048 | 0.017 |
|  | $\delta$ | 0.963 | 0.013 | 0.021 | 0.004 |
| TMB with errors and response with misclassification  e ~ Laplace (0, ${1.5}^{2}$)  $\left( \eta,\delta\right)=\left( 0.75,0.98 \right)$  Naive estimator | $\lambda$ | 0.928 | -0.072 | 0.067 | 0.081 |
|  | $\beta$ | 0.604 | -0.396 | 0.168 | 0.178 |
|  | $\beta_{m}$ | -0.189 | 0.211 | 0.041 | 0.048 |
|  | $\alpha$ | -1.065 | -0.265 | 0.304 | 0.266 |
|  | $\alpha_{m}$ | 0.121 | -0.279 | 0.073 | 0.066 |
|  | $\sigma_{b}$ | 0.418 | -0.082 | 0.121 | 0.183 |
| TMB with errors and response with misclassification  e ~ Laplace (0, ${1.5}^{2}$)  $\left( \eta,\delta\right)=\left( 0.75,0.98 \right)$  Bayesian estimator | $\lambda$ | 1.021 | 0.021 | 0.091 | 0.123 |
|  | $\beta$ | 0.945 | -0.055 | 0.280 | 0.314 |
|  | $\beta_{m}$ | -0.399 | 0.001 | 0.127 | 0.158 |
|  | $\alpha$ | -0.790 | 0.010 | 0.625 | 0.394 |
|  | $\alpha_{m}$ | 0.403 | 0.003 | 0.303 | 0.193 |
|  | $\sigma_{b}$ | 0.503 | 0.003 | 0.155 | 0.258 |
|  | $\eta$ | 0.735 | -0.015 | 0.048 | 0.017 |
|  | $\delta$ | 0.963 | -0.017 | 0.022 | 0.004 |
| TMB with errors and response with misclassification  e ~ Extreme (0, ${1.0}^{2}$)  $\left( \eta,\delta\right)=\left( 0.70,0.95 \right)$  Naive estimator | $\lambda$ | 0.948 | -0.052 | 0.044 | 0.054 |
|  | $\beta$ | 0.733 | -0.267 | 0.110 | 0.120 |
|  | $\beta_{m}$ | -0.261 | 0.139 | 0.031 | 0.035 |
|  | $\alpha$ | -1.170 | -0.370 | 0.198 | 0.184 |
|  | $\alpha_{m}$ | 0.142 | -0.258 | 0.053 | 0.051 |
|  | $\sigma_{b}$ | 0.447 | -0.053 | 0.083 | 0.124 |
| TMB with errors and response with misclassification  e ~ Extreme (0, ${1.0}^{2}$)  $\left( \eta,\delta\right)=\left( 0.70,0.95 \right)$  Bayesian estimator | $\lambda$ | 1.034 | 0.034 | 0.091 | 0.111 |
|  | $\beta$ | 1.014 | 0.014 | 0.270 | 0.291 |
|  | $\beta_{m}$ | -0.456 | -0.056 | 0.128 | 0.137 |
|  | $\alpha$ | -0.760 | 0.040 | 0.595 | 0.390 |
|  | $\alpha_{m}$ | 0.409 | 0.009 | 0.288 | 0.176 |
|  | $\sigma_{b}$ | 0.489 | -0.011 | 0.152 | 0.248 |
|  | $\eta$ | 0.732 | 0.032 | 0.049 | 0.016 |
|  | $\delta$ | 0.964 | 0.014 | 0.021 | 0.004 |
| TMB with errors and response with misclassification  e ~ Extreme (0, ${1.0}^{2}$)  $\left( \eta,\delta\right)=\left( 0.75,0.98 \right)$  Naive estimator | $\lambda$ | 0.955 | -0.045 | 0.069 | 0.081 |
|  | $\beta$ | 0.743 | -0.257 | 0.173 | 0.194 |
|  | $\beta_{m}$ | -0.263 | 0.137 | 0.049 | 0.052 |
|  | $\alpha$ | -1.124 | -0.324 | 0.314 | 0.266 |
|  | $\alpha_{m}$ | 0.165 | -0.235 | 0.086 | 0.081 |
|  | $\sigma_{b}$ | 0.434 | -0.066 | 0.122 | 0.183 |
| TMB with errors and response with misclassification  e ~ Extreme (0, ${1.0}^{2}$)  $\left( \eta,\delta\right)=\left( 0.75,0.98 \right)$  Bayesian estimator | $\lambda$ | 1.048 | 0.048 | 0.093 | 0.124 |
|  | $\beta$ | 1.021 | 0.021 | 0.272 | 0.305 |
|  | $\beta_{m}$ | -0.452 | -0.052 | 0.129 | 0.133 |
|  | $\alpha$ | -0.808 | -0.008 | 0.602 | 0.397 |
|  | $\alpha_{m}$ | 0.434 | 0.034 | 0.289 | 0.179 |
|  | $\sigma_{b}$ | 0.508 | 0.008 | 0.157 | 0.260 |
|  | $\eta$ | 0.737 | -0.013 | 0.048 | 0.018 |
|  | $\delta$ | 0.963 | -0.017 | 0.021 | 0.004 |
| TMB with errors and response with misclassification  e ~ Extreme (0, ${1.5}^{2}$)  $\left( \eta,\delta\right)=\left( 0.70,0.95 \right)$  Naive estimator | $\lambda$ | 0.930 | -0.070 | 0.044 | 0.051 |
|  | $\beta$ | 0.600 | -0.400 | 0.107 | 0.119 |
|  | $\beta_{m}$ | -0.183 | 0.217 | 0.025 | 0.030 |
|  | $\alpha$ | -1.134 | -0.334 | 0.192 | 0.178 |
|  | $\alpha_{m}$ | 0.107 | -0.293 | 0.046 | 0.046 |
|  | $\sigma_{b}$ | 0.458 | -0.042 | 0.085 | 0.125 |
| TMB with errors and response with misclassification  e ~ Extreme (0, ${1.5}^{2}$)  $\left( \eta,\delta\right)=\left( 0.70,0.95 \right)$  Bayesian estimator | $\lambda$ | 1.021 | 0.021 | 0.092 | 0.110 |
|  | $\beta$ | 0.952 | -0.048 | 0.281 | 0.325 |
|  | $\beta_{m}$ | -0.415 | -0.015 | 0.136 | 0.159 |
|  | $\alpha$ | -0.744 | 0.056 | 0.608 | 0.402 |
|  | $\alpha_{m}$ | 0.368 | -0.032 | 0.303 | 0.195 |
|  | $\sigma_{b}$ | 0.477 | -0.023 | 0.151 | 0.248 |
|  | $\eta$ | 0.735 | 0.035 | 0.048 | 0.016 |
|  | $\delta$ | 0.963 | 0.013 | 0.021 | 0.004 |
| TMB with errors and response with misclassification  e ~ Extreme (0, ${1.5}^{2}$)  $\left( \eta,\delta\right)=\left( 0.75,0.98 \right)$  Naive estimator | $\lambda$ | 0.931 | -0.069 | 0.067 | 0.079 |
|  | $\beta$ | 0.602 | -0.398 | 0.168 | 0.189 |
|  | $\beta_{m}$ | -0.184 | 0.216 | 0.040 | 0.049 |
|  | $\alpha$ | -1.086 | -0.289 | 0.305 | 0.272 |
|  | $\alpha_{m}$ | 0.133 | -0.267 | 0.073 | 0.067 |
|  | $\sigma_{b}$ | 0.436 | -0.064 | 0.122 | 0.189 |
| TMB with errors and response with misclassification  e ~ Extreme (0, ${1.5}^{2}$)  $\left( \eta,\delta\right)=\left( 0.75,0.98 \right)$  Bayesian estimator | $\lambda$ | 1.019 | 0.019 | 0.094 | 0.114 |
|  | $\beta$ | 0.907 | -0.093 | 0.281 | 0.316 |
|  | $\beta_{m}$ | -0.402 | -0.002 | 0.138 | 0.164 |
|  | $\alpha$ | -0.751 | 0.049 | 0.621 | 0.402 |
|  | $\alpha_{m}$ | 0.391 | -0.009 | 0.312 | 0.185 |
|  | $\sigma_{b}$ | 0.485 | -0.015 | 0.157 | 0.247 |
|  | $\eta$ | 0.737 | -0.013 | 0.048 | 0.016 |
|  | $\delta$ | 0.963 | -0.017 | 0.022 | 0.004 |
| TMB ~ Extreme (mean = 1, var = ${1.5}^{2}$) | | | | | |
| True-data  Bayesian estimator | $\lambda$ | 0.998 | -0.002 | 0.048 | 0.058 |
|  | $\beta$ | 0.995 | -0.005 | 0.119 | 0.136 |
|  | $\beta_{m}$ | -0.401 | -0.001 | 0.039 | 0.044 |
|  | $\alpha$ | -0.815 | -0.015 | 0.205 | 0.206 |
|  | $\alpha_{m}$ | 0.404 | 0.004 | 0.069 | 0.072 |
|  | $\sigma_{b}$ | 0.475 | -0.025 | 0.089 | 0.144 |
| TMB with errors  e ~ Normal (0, ${1.0}^{2}$)  Naive estimator | $\lambda$ | 0.953 | -0.047 | 0.070 | 0.086 |
|  | $\beta$ | 0.735 | -0.265 | 0.176 | 0.197 |
|  | $\beta_{m}$ | -0.273 | 0.127 | 0.051 | 0.055 |
|  | $\alpha$ | -0.625 | 0.175 | 0.304 | 0.276 |
|  | $\alpha_{m}$ | 0.285 | -0.115 | 0.088 | 0.087 |
|  | $\sigma_{b}$ | 0.431 | -0.069 | 0.126 | 0.194 |
| TMB with errors  e ~ Normal (0, ${1.0}^{2}$)  Bayesian estimator | $\lambda$ | 1.037 | 0.037 | 0.091 | 0.109 |
|  | $\beta$ | 1.031 | 0.031 | 0.278 | 0.311 |
|  | $\beta_{m}$ | -0.486 | -0.086 | 0.140 | 0.125 |
|  | $\alpha$ | -0.774 | 0.026 | 0.396 | 0.338 |
|  | $\alpha_{m}$ | 0.465 | 0.065 | 0.194 | 0.154 |
|  | $\sigma_{b}$ | 0.481 | -0.019 | 0.142 | 0.236 |
| TMB with errors  e ~ Normal (0, ${1.0}^{2}$)  Corrected score estimator | $\lambda$ | 1.011 | 0.011 | 0.057 | 0.097 |
|  | $\beta$ | 0.975 | -0.025 | 0.152 | 0.260 |
|  | $\beta_{m}$ | -0.399 | 0.001 | 0.052 | 0.105 |
|  | $\alpha$ | -0.750 | 0.050 | 0.332 | 0.379 |
|  | $\alpha_{m}$ | 0.374 | -0.026 | 0.114 | 0.152 |
|  | $\sigma_{b}$ | 0.506 | 0.006 | 0.045 | 0.191 |
| TMB with errors  e ~ Normal (0, ${1.5}^{2}$)  Naive estimator | $\lambda$ | 0.918 | -0.082 | 0.066 | 0.080 |
|  | $\beta$ | 0.588 | -0.412 | 0.167 | 0.187 |
|  | $\beta_{m}$ | -0.190 | 0.210 | 0.042 | 0.046 |
|  | $\alpha$ | -0.519 | 0.281 | 0.291 | 0.258 |
|  | $\alpha_{m}$ | 0.208 | -0.192 | 0.073 | 0.076 |
|  | $\sigma_{b}$ | 0.408 | -0.092 | 0.119 | 0.188 |
| TMB with errors  e ~ Normal (0, ${1.5}^{2}$)  Bayesian estimator | $\lambda$ | 1.021 | 0.021 | 0.091 | 0.106 |
|  | $\beta$ | 0.987 | -0.013 | 0.297 | 0.322 |
|  | $\beta_{m}$ | -0.454 | -0.054 | 0.152 | 0.145 |
|  | $\alpha$ | -0.740 | 0.060 | 0.407 | 0.353 |
|  | $\alpha_{m}$ | 0.412 | 0.012 | 0.204 | 0.165 |
|  | $\sigma_{b}$ | 0.443 | -0.057 | 0.133 | 0.226 |
| TMB with errors  e ~ Normal (0, ${1.5}^{2}$)  Corrected score estimator | $\lambda$ | 0.958 | -0.042 | 0.055 | 0.105 |
|  | $\beta$ | 0.828 | -0.172 | 0.155 | 0.302 |
|  | $\beta_{m}$ | -0.304 | 0.096 | 0.051 | 0.158 |
|  | $\alpha$ | -0.788 | 0.012 | 0.355 | 0.422 |
|  | $\alpha_{m}$ | 0.404 | 0.004 | 0.104 | 0.183 |
|  | $\sigma_{b}$ | 0.430 | -0.070 | 0.043 | 0.191 |
| TMB with errors  e ~ Laplace (0, ${1.0}^{2}$)  Naive estimator | $\lambda$ | 0.958 | -0.042 | 0.071 | 0.081 |
|  | $\beta$ | 0.762 | -0.238 | 0.178 | 0.212 |
|  | $\beta_{m}$ | -0.272 | 0.128 | 0.051 | 0.058 |
|  | $\alpha$ | -0.639 | 0.161 | 0.308 | 0.291 |
|  | $\alpha_{m}$ | 0.292 | -0.108 | 0.090 | 0.088 |
|  | $\sigma_{b}$ | 0.455 | -0.045 | 0.127 | 0.202 |
| TMB with errors  e ~ Laplace (0, ${1.0}^{2}$)  Bayesian estimator | $\lambda$ | 1.026 | 0.026 | 0.089 | 0.102 |
|  | $\beta$ | 1.006 | 0.006 | 0.277 | 0.298 |
|  | $\beta_{m}$ | -0.473 | -0.073 | 0.135 | 0.135 |
|  | $\alpha$ | -0.756 | 0.044 | 0.393 | 0.361 |
|  | $\alpha_{m}$ | 0.458 | 0.058 | 0.190 | 0.192 |
|  | $\sigma_{b}$ | 0.461 | -0.039 | 0.140 | 0.281 |
| TMB with errors  e ~ Laplace (0, ${1.0}^{2}$)  Corrected score estimator | $\lambda$ | 1.012 | 0.012 | 0.057 | 0.098 |
|  | $\beta$ | 0.987 | -0.013 | 0.151 | 0.278 |
|  | $\beta_{m}$ | -0.398 | 0.002 | 0.051 | 0.127 |
|  | $\alpha$ | -0.745 | 0.055 | 0.326 | 0.384 |
|  | $\alpha_{m}$ | 0.364 | -0.036 | 0.107 | 0.160 |
|  | $\sigma_{b}$ | 0.523 | 0.023 | 0.045 | 0.220 |
| TMB with errors  e ~ Laplace (0, ${1.5}^{2}$)  Naive estimator | $\lambda$ | 0.926 | -0.074 | 0.067 | 0.082 |
|  | $\beta$ | 0.606 | -0.394 | 0.169 | 0.184 |
|  | $\beta_{m}$ | -0.190 | 0.210 | 0.042 | 0.049 |
|  | $\alpha$ | -0.533 | 0.267 | 0.292 | 0.261 |
|  | $\alpha_{m}$ | 0.214 | -0.186 | 0.074 | 0.080 |
|  | $\sigma_{b}$ | 0.425 | -0.075 | 0.122 | 0.191 |
| TMB with errors  e ~ Laplace (0, ${1.5}^{2}$)  Bayesian estimator | $\lambda$ | 1.008 | 0.008 | 0.089 | 0.104 |
|  | $\beta$ | 0.955 | -0.045 | 0.285 | 0.326 |
|  | $\beta_{m}$ | -0.417 | -0.017 | 0.139 | 0.152 |
|  | $\alpha$ | -0.725 | 0.075 | 0.401 | 0.340 |
|  | $\alpha_{m}$ | 0.401 | 0.001 | 0.191 | 0.169 |
|  | $\sigma_{b}$ | 0.450 | -0.050 | 0.135 | 0.224 |
| TMB with errors  e ~ Laplace (0, ${1.5}^{2}$)  Corrected score estimator | $\lambda$ | 0.978 | -0.022 | 0.055 | 0.115 |
|  | $\beta$ | 0.817 | -0.183 | 0.151 | 0.361 |
|  | $\beta_{m}$ | -0.308 | 0.092 | 0.045 | 0.157 |
|  | $\alpha$ | -0.842 | -0.042 | 0.400 | 0.417 |
|  | $\alpha_{m}$ | 0.435 | 0.035 | 0.180 | 0.198 |
|  | $\sigma_{b}$ | 0.478 | -0.022 | 0.043 | 0.195 |
| TMB with errors  e ~ Extreme (0, ${1.0}^{2}$)  Naive estimator | $\lambda$ | 0.967 | -0.033 | 0.072 | 0.086 |
|  | $\beta$ | 0.750 | -0.250 | 0.180 | 0.191 |
|  | $\beta_{m}$ | -0.266 | 0.134 | 0.051 | 0.054 |
|  | $\alpha$ | -0.627 | 0.173 | 0.309 | 0.285 |
|  | $\alpha_{m}$ | 0.289 | -0.111 | 0.089 | 0.087 |
|  | $\sigma_{b}$ | 0.469 | -0.031 | 0.129 | 0.200 |
| TMB with errors  e ~ Extreme (0, ${1.0}^{2}$)  Bayesian estimator | $\lambda$ | 1.031 | 0.031 | 0.088 | 0.108 |
|  | $\beta$ | 0.990 | -0.010 | 0.271 | 0.309 |
|  | $\beta_{m}$ | -0.457 | -0.057 | 0.131 | 0.133 |
|  | $\alpha$ | -0.770 | 0.030 | 0.388 | 0.335 |
|  | $\alpha_{m}$ | 0.454 | 0.054 | 0.185 | 0.154 |
|  | $\sigma_{b}$ | 0.477 | -0.023 | 0.139 | 0.225 |
| TMB with errors  e ~ Extreme (0, ${1.0}^{2}$)  Corrected score estimator | $\lambda$ | 1.017 | 0.017 | 0.057 | 0.109 |
|  | $\beta$ | 0.955 | -0.045 | 0.149 | 0.275 |
|  | $\beta_{m}$ | -0.389 | 0.011 | 0.048 | 0.123 |
|  | $\alpha$ | -0.773 | 0.027 | 0.324 | 0.374 |
|  | $\alpha_{m}$ | 0.354 | -0.046 | 0.107 | 0.165 |
|  | $\sigma_{b}$ | 0.536 | 0.036 | 0.046 | 0.208 |
| TMB with errors  e ~ Extreme (0, ${1.5}^{2}$)  Naive estimator | $\lambda$ | 0.931 | -0.069 | 0.068 | 0.086 |
|  | $\beta$ | 0.592 | -0.408 | 0.170 | 0.202 |
|  | $\beta_{m}$ | -0.186 | 0.214 | 0.041 | 0.051 |
|  | $\alpha$ | -0.540 | 0.260 | 0.295 | 0.284 |
|  | $\alpha_{m}$ | 0.212 | -0.188 | 0.075 | 0.077 |
|  | $\sigma_{b}$ | 0.433 | -0.067 | 0.121 | 0.206 |
| TMB with errors  e ~ Extreme (0, ${1.5}^{2}$)  Bayesian estimator | $\lambda$ | 1.001 | 0.001 | 0.087 | 0.106 |
|  | $\beta$ | 0.900 | -0.100 | 0.280 | 0.311 |
|  | $\beta_{m}$ | -0.407 | -0.007 | 0.137 | 0.165 |
|  | $\alpha$ | -0.720 | 0.080 | 0.400 | 0.347 |
|  | $\alpha_{m}$ | 0.405 | 0.005 | 0.196 | 0.170 |
|  | $\sigma_{b}$ | 0.440 | -0.060 | 0.133 | 0.215 |
| TMB with errors  e ~ Extreme (0, ${1.5}^{2}$)  Corrected score estimator | $\lambda$ | 0.956 | -0.044 | 0.053 | 0.105 |
|  | $\beta$ | 0.708 | -0.292 | 0.144 | 0.308 |
|  | $\beta_{m}$ | -0.262 | 0.138 | 0.039 | 0.124 |
|  | $\alpha$ | -0.777 | 0.023 | 0.349 | 0.431 |
|  | $\alpha_{m}$ | 0.377 | -0.023 | 0.114 | 0.180 |
|  | $\sigma_{b}$ | 0.461 | -0.039 | 0.043 | 0.205 |
| Response with misclassification  $\left( \eta,\delta\right)=\left( 0.70,0.95 \right)$  Naive estimator | $\lambda$ | 0.985 | -0.015 | 0.045 | 0.052 |
|  | $\beta$ | 0.970 | -0.030 | 0.115 | 0.128 |
|  | $\beta_{m}$ | -0.394 | 0.006 | 0.038 | 0.039 |
|  | $\alpha$ | -1.255 | -0.455 | 0.207 | 0.179 |
|  | $\alpha_{m}$ | 0.205 | -0.195 | 0.064 | 0.065 |
|  | $\sigma_{b}$ | 0.416 | -0.084 | 0.082 | 0.129 |
| Response with misclassification  $\left( \eta,\delta\right)=\left( 0.70,0.95 \right)$  Bayesian estimator | $\lambda$ | 1.011 | 0.011 | 0.052 | 0.068 |
|  | $\beta$ | 1.010 | 0.010 | 0.125 | 0.148 |
|  | $\beta_{m}$ | -0.403 | -0.003 | 0.040 | 0.044 |
|  | $\alpha$ | -0.835 | -0.035 | 0.361 | 0.335 |
|  | $\alpha_{m}$ | 0.394 | -0.006 | 0.117 | 0.113 |
|  | $\sigma_{b}$ | 0.497 | -0.003 | 0.100 | 0.161 |
|  | $\eta$ | 0.729 | 0.029 | 0.044 | 0.024 |
|  | $\delta$ | 0.964 | 0.014 | 0.020 | 0.006 |
| Response with misclassification  $\left( \eta,\delta\right)=\left( 0.75,0.98 \right)$  Naive estimator | $\lambda$ | 0.981 | -0.019 | 0.045 | 0.054 |
|  | $\beta$ | 0.977 | -0.023 | 0.115 | 0.128 |
|  | $\beta_{m}$ | -0.393 | 0.007 | 0.038 | 0.041 |
|  | $\alpha$ | -1.271 | -0.471 | 0.207 | 0.178 |
|  | $\alpha_{m}$ | 0.240 | -0.160 | 0.064 | 0.065 |
|  | $\sigma_{b}$ | 0.426 | -0.074 | 0.084 | 0.134 |
| Response with misclassification  $\left( \eta,\delta\right)=\left( 0.75,0.98 \right)$  Bayesian estimator | $\lambda$ | 1.014 | 0.014 | 0.052 | 0.070 |
|  | $\beta$ | 1.009 | 0.009 | 0.126 | 0.149 |
|  | $\beta_{m}$ | -0.404 | -0.004 | 0.040 | 0.046 |
|  | $\alpha$ | -0.877 | -0.077 | 0.366 | 0.343 |
|  | $\alpha_{m}$ | 0.458 | 0.058 | 0.126 | 0.120 |
|  | $\sigma_{b}$ | 0.508 | 0.008 | 0.101 | 0.164 |
|  | $\eta$ | 0.732 | -0.018 | 0.044 | 0.023 |
|  | $\delta$ | 0.964 | -0.016 | 0.020 | 0.006 |
| TMB with errors and response with misclassification  e ~ Normal (0, ${1.0}^{2}$)  $\left( \eta,\delta\right)=\left( 0.70,0.95 \right)$  Naive estimator | $\lambda$ | 0.941 | -0.059 | 0.044 | 0.051 |
|  | $\beta$ | 0.731 | -0.269 | 0.110 | 0.124 |
|  | $\beta_{m}$ | -0.268 | 0.132 | 0.032 | 0.034 |
|  | $\alpha$ | -1.172 | -0.372 | 0.196 | 0.181 |
|  | $\alpha_{m}$ | 0.140 | -0.260 | 0.052 | 0.055 |
|  | $\sigma_{b}$ | 0.430 | -0.070 | 0.083 | 0.129 |
| TMB with errors and response with misclassification  e ~ Normal (0, ${1.0}^{2}$)  $\left( \eta,\delta\right)=\left( 0.70,0.95 \right)$  Bayesian estimator | $\lambda$ | 1.037 | 0.037 | 0.094 | 0.111 |
|  | $\beta$ | 1.018 | 0.018 | 0.293 | 0.314 |
|  | $\beta_{m}$ | -0.492 | -0.092 | 0.146 | 0.132 |
|  | $\alpha$ | -0.789 | 0.011 | 0.605 | 0.397 |
|  | $\alpha_{m}$ | 0.427 | 0.027 | 0.303 | 0.183 |
|  | $\sigma_{b}$ | 0.488 | -0.012 | 0.153 | 0.257 |
|  | $\eta$ | 0.734 | 0.034 | 0.046 | 0.016 |
|  | $\delta$ | 0.964 | 0.014 | 0.021 | 0.004 |
| TMB with errors and response with misclassification  e ~ Normal (0, ${1.0}^{2}$)  $\left( \eta,\delta\right)=\left( 0.75,0.98 \right)$  Naive estimator | $\lambda$ | 0.944 | -0.056 | 0.044 | 0.054 |
|  | $\beta$ | 0.741 | -0.259 | 0.110 | 0.123 |
|  | $\beta_{m}$ | -0.268 | 0.132 | 0.032 | 0.034 |
|  | $\alpha$ | -1.145 | -0.345 | 0.197 | 0.188 |
|  | $\alpha_{m}$ | 0.170 | -0.230 | 0.054 | 0.050 |
|  | $\sigma_{b}$ | 0.437 | -0.063 | 0.084 | 0.124 |
| TMB with errors and response with misclassification  e ~ Normal (0, ${1.0}^{2}$)  $\left( \eta,\delta\right)=\left( 0.75,0.98 \right)$  Bayesian estimator | $\lambda$ | 1.038 | 0.038 | 0.093 | 0.115 |
|  | $\beta$ | 0.995 | -0.005 | 0.286 | 0.309 |
|  | $\beta_{m}$ | -0.483 | -0.083 | 0.137 | 0.121 |
|  | $\alpha$ | -0.756 | 0.044 | 0.599 | 0.399 |
|  | $\alpha_{m}$ | 0.456 | 0.056 | 0.303 | 0.173 |
|  | $\sigma_{b}$ | 0.479 | -0.021 | 0.154 | 0.254 |
|  | $\eta$ | 0.735 | -0.015 | 0.046 | 0.016 |
|  | $\delta$ | 0.964 | -0.016 | 0.021 | 0.004 |
| TMB with errors and response with misclassification  e ~ Normal (0, ${1.5}^{2}$)  $\left( \eta,\delta\right)=\left( 0.70,0.95 \right)$  Naive estimator | $\lambda$ | 0.918 | -0.082 | 0.043 | 0.053 |
|  | $\beta$ | 0.588 | -0.412 | 0.106 | 0.117 |
|  | $\beta_{m}$ | -0.189 | 0.211 | 0.026 | 0.028 |
|  | $\alpha$ | -1.112 | -0.312 | 0.190 | 0.184 |
|  | $\alpha_{m}$ | 0.107 | -0.293 | 0.044 | 0.043 |
|  | $\sigma_{b}$ | 0.430 | -0.070 | 0.083 | 0.131 |
| TMB with errors and response with misclassification  e ~ Normal (0, ${1.5}^{2}$)  $\left( \eta,\delta\right)=\left( 0.70,0.95 \right)$  Bayesian estimator | $\lambda$ | 1.041 | 0.041 | 0.098 | 0.114 |
|  | $\beta$ | 0.985 | -0.015 | 0.304 | 0.322 |
|  | $\beta_{m}$ | -0.465 | -0.065 | 0.158 | 0.145 |
|  | $\alpha$ | -0.739 | 0.061 | 0.612 | 0.388 |
|  | $\alpha_{m}$ | 0.396 | -0.004 | 0.329 | 0.180 |
|  | $\sigma_{b}$ | 0.492 | -0.008 | 0.159 | 0.248 |
|  | $\eta$ | 0.733 | 0.033 | 0.046 | 0.016 |
|  | $\delta$ | 0.964 | 0.014 | 0.021 | 0.003 |
| TMB with errors and response with misclassification  e ~ Normal (0, ${1.5}^{2}$)  $\left( \eta,\delta\right)=\left( 0.75,0.98 \right)$  Naive estimator | $\lambda$ | 0.918 | -0.082 | 0.043 | 0.051 |
|  | $\beta$ | 0.594 | -0.406 | 0.105 | 0.111 |
|  | $\beta_{m}$ | -0.190 | 0.210 | 0.026 | 0.028 |
|  | $\alpha$ | -1.060 | -0.260 | 0.189 | 0.186 |
|  | $\alpha_{m}$ | 0.120 | -0.280 | 0.045 | 0.045 |
|  | $\sigma_{b}$ | 0.429 | -0.071 | 0.083 | 0.123 |
| TMB with errors and response with misclassification  e ~ Normal (0, ${1.5}^{2}$)  $\left( \eta,\delta\right)=\left( 0.75,0.98 \right)$  Bayesian estimator | $\lambda$ | 1.023 | 0.023 | 0.094 | 0.113 |
|  | $\beta$ | 0.999 | -0.001 | 0.297 | 0.333 |
|  | $\beta_{m}$ | -0.458 | -0.058 | 0.161 | 0.143 |
|  | $\alpha$ | -0.726 | 0.074 | 0.634 | 0.388 |
|  | $\alpha_{m}$ | 0.416 | 0.016 | 0.336 | 0.190 |
|  | $\sigma_{b}$ | 0.461 | -0.039 | 0.149 | 0.268 |
|  | $\eta$ | 0.735 | -0.015 | 0.049 | 0.015 |
|  | $\delta$ | 0.964 | -0.016 | 0.021 | 0.003 |
| TMB with errors and response with misclassification  e ~ Laplace (0, ${1.0}^{2}$)  $\left( \eta,\delta\right)=\left( 0.70,0.95 \right)$  Naive estimator | $\lambda$ | 0.946 | -0.054 | 0.044 | 0.051 |
|  | $\beta$ | 0.736 | -0.264 | 0.110 | 0.117 |
|  | $\beta_{m}$ | -0.270 | 0.130 | 0.032 | 0.034 |
|  | $\alpha$ | -1.169 | -0.369 | 0.197 | 0.175 |
|  | $\alpha_{m}$ | 0.145 | -0.255 | 0.053 | 0.052 |
|  | $\sigma_{b}$ | 0.444 | -0.056 | 0.084 | 0.132 |
| TMB with errors and response with misclassification  e ~ Laplace (0, ${1.0}^{2}$)  $\left( \eta,\delta\right)=\left( 0.70,0.95 \right)$  Bayesian estimator | $\lambda$ | 1.035 | 0.035 | 0.091 | 0.109 |
|  | $\beta$ | 0.996 | -0.004 | 0.274 | 0.289 |
|  | $\beta_{m}$ | -0.475 | -0.075 | 0.134 | 0.135 |
|  | $\alpha$ | -0.801 | -0.001 | 0.595 | 0.393 |
|  | $\alpha_{m}$ | 0.429 | 0.029 | 0.295 | 0.185 |
|  | $\sigma_{b}$ | 0.472 | -0.028 | 0.150 | 0.238 |
|  | $\eta$ | 0.734 | 0.034 | 0.049 | 0.017 |
|  | $\delta$ | 0.964 | 0.014 | 0.021 | 0.004 |
| TMB with errors and response with misclassification  e ~ Laplace (0, ${1.0}^{2}$)  $\left( \eta,\delta\right)=\left( 0.75,0.98 \right)$  Naive estimator | $\lambda$ | 0.948 | -0.052 | 0.044 | 0.052 |
|  | $\beta$ | 0.748 | -0.252 | 0.110 | 0.120 |
|  | $\beta_{m}$ | -0.269 | 0.131 | 0.032 | 0.035 |
|  | $\alpha$ | -1.149 | -0.349 | 0.197 | 0.187 |
|  | $\alpha_{m}$ | 0.168 | -0.232 | 0.054 | 0.053 |
|  | $\sigma_{b}$ | 0.446 | -0.054 | 0.083 | 0.129 |
| TMB with errors and response with misclassification  e ~ Laplace (0, ${1.0}^{2}$)  $\left( \eta,\delta\right)=\left( 0.75,0.98 \right)$  Bayesian estimator | $\lambda$ | 1.050 | 0.050 | 0.093 | 0.125 |
|  | $\beta$ | 1.001 | 0.001 | 0.280 | 0.313 |
|  | $\beta_{m}$ | -0.470 | -0.070 | 0.131 | 0.131 |
|  | $\alpha$ | -0.772 | 0.028 | 0.610 | 0.396 |
|  | $\alpha_{m}$ | 0.470 | 0.070 | 0.306 | 0.183 |
|  | $\sigma_{b}$ | 0.496 | -0.004 | 0.153 | 0.269 |
|  | $\eta$ | 0.734 | -0.016 | 0.048 | 0.016 |
|  | $\delta$ | 0.963 | -0.017 | 0.021 | 0.004 |
| TMB with errors and response with misclassification  e ~ Laplace (0, ${1.5}^{2}$)  $\left( \eta,\delta\right)=\left( 0.70,0.95 \right)$  Naive estimator | $\lambda$ | 0.917 | -0.083 | 0.042 | 0.051 |
|  | $\beta$ | 0.592 | -0.403 | 0.105 | 0.116 |
|  | $\beta_{m}$ | -0.189 | 0.211 | 0.026 | 0.029 |
|  | $\alpha$ | -1.108 | -0.308 | 0.190 | 0.188 |
|  | $\alpha_{m}$ | 0.106 | -0.294 | 0.045 | 0.042 |
|  | $\sigma_{b}$ | 0.430 | --0.070 | 0.081 | 0.127 |
| TMB with errors and response with misclassification  e ~ Laplace (0, ${1.5}^{2}$)  $\left( \eta,\delta\right)=\left( 0.70,0.95 \right)$  Bayesian estimator | $\lambda$ | 1.025 | 0.025 | 0.094 | 0.117 |
|  | $\beta$ | 0.949 | -0.051 | 0.284 | 0.331 |
|  | $\beta_{m}$ | -0.415 | -0.015 | 0.135 | 0.161 |
|  | $\alpha$ | -0.728 | 0.072 | 0.608 | 0.393 |
|  | $\alpha_{m}$ | 0.368 | -0.032 | 0.305 | 0.199 |
|  | $\sigma_{b}$ | 0.473 | -0.027 | 0.154 | 0.260 |
|  | $\eta$ | 0.733 | 0.033 | 0.049 | 0.016 |
|  | $\delta$ | 0.964 | 0.014 | 0.021 | 0.004 |
| TMB with errors and response with misclassification  e ~ Laplace (0, ${1.5}^{2}$)  $\left( \eta,\delta\right)=\left( 0.75,0.98 \right)$  Naive estimator | $\lambda$ | 0.919 | -0.081 | 0.042 | 0.052 |
|  | $\beta$ | 0.600 | -0.400 | 0.105 | 0.117 |
|  | $\beta_{m}$ | -0.189 | 0.211 | 0.026 | 0.030 |
|  | $\alpha$ | -1.073 | -0.273 | 0.189 | 0.184 |
|  | $\alpha_{m}$ | 0.120 | -0.280 | 0.045 | 0.045 |
|  | $\sigma_{b}$ | 0.427 | -0.073 | 0.082 | 0.126 |
| TMB with errors and response with misclassification  e ~ Laplace (0, ${1.5}^{2}$)  $\left( \eta,\delta\right)=\left( 0.75,0.98 \right)$  Bayesian estimator | $\lambda$ | 1.031 | 0.031 | 0.096 | 0.112 |
|  | $\beta$ | 0.953 | -0.047 | 0.292 | 0.325 |
|  | $\beta_{m}$ | -0.419 | -0.019 | 0.140 | 0.146 |
|  | $\alpha$ | -0.747 | 0.053 | 0.632 | 0.384 |
|  | $\alpha_{m}$ | 0.421 | 0.021 | 0.326 | 0.182 |
|  | $\sigma_{b}$ | 0.498 | -0.002 | 0.156 | 0.258 |
|  | $\eta$ | 0.735 | -0.015 | 0.048 | 0.017 |
|  | $\delta$ | 0.963 | -0.017 | 0.021 | 0.004 |
| TMB with errors and response with misclassification  e ~ Extreme (0, ${1.0}^{2}$)  $\left( \eta,\delta\right)=\left( 0.70,0.95 \right)$  Naive estimator | $\lambda$ | 0.943 | -0.057 | 0.044 | 0.050 |
|  | $\beta$ | 0.725 | -0.275 | 0.110 | 0.124 |
|  | $\beta_{m}$ | -0.259 | 0.141 | 0.031 | 0.036 |
|  | $\alpha$ | -1.131 | -0.331 | 0.197 | 0.188 |
|  | $\alpha_{m}$ | 0.139 | -0.261 | 0.054 | 0.051 |
|  | $\sigma_{b}$ | 0.436 | -0.064 | 0.084 | 0.125 |
| TMB with errors and response with misclassification  e ~ Extreme (0, ${1.0}^{2}$)  $\left( \eta,\delta\right)=\left( 0.70,0.95 \right)$  Bayesian estimator | $\lambda$ | 1.042 | 0.042 | 0.094 | 0.118 |
|  | $\beta$ | 1.018 | 0.018 | 0.282 | 0.319 |
|  | $\beta_{m}$ | -0.468 | -0.068 | 0.133 | 0.140 |
|  | $\alpha$ | -0.786 | 0.014 | 0.601 | 0.386 |
|  | $\alpha_{m}$ | 0.421 | 0.021 | 0.294 | 0.187 |
|  | $\sigma_{b}$ | 0.494 | -0.006 | 0.156 | 0.259 |
|  | $\eta$ | 0.734 | 0.034 | 0.048 | 0.016 |
|  | $\delta$ | 0.964 | 0.014 | 0.021 | 0.004 |
| TMB with errors and response with misclassification  e ~ Extreme (0, ${1.0}^{2}$)  $\left( \eta,\delta\right)=\left( 0.75,0.98 \right)$  Naive estimator | $\lambda$ | 0.948 | -0.052 | 0.045 | 0.051 |
|  | $\beta$ | 0.739 | -0.261 | 0.111 | 0.121 |
|  | $\beta_{m}$ | -0.264 | 0.136 | 0.031 | 0.035 |
|  | $\alpha$ | -1.169 | -0.369 | 0.198 | 0.188 |
|  | $\alpha_{m}$ | 0.171 | -0.229 | 0.054 | 0.053 |
|  | $\sigma_{b}$ | 0.453 | -0.047 | 0.086 | 0.121 |
| TMB with errors and response with misclassification  e ~ Extreme (0, ${1.0}^{2}$)  $\left( \eta,\delta\right)=\left( 0.75,0.98 \right)$  Bayesian estimator | $\lambda$ | 1.034 | 0.034 | 0.091 | 0.118 |
|  | $\beta$ | 0.959 | -0.041 | 0.272 | 0.307 |
|  | $\beta_{m}$ | -0.460 | -0.060 | 0.128 | 0.127 |
|  | $\alpha$ | -0.781 | 0.019 | 0.610 | 0.399 |
|  | $\alpha_{m}$ | 0.458 | 0.058 | 0.299 | 0.182 |
|  | $\sigma_{b}$ | 0.483 | -0.017 | 0.150 | 0.271 |
|  | $\eta$ | 0.736 | -0.014 | 0.048 | 0.016 |
|  | $\delta$ | 0.963 | -0.017 | 0.021 | 0.004 |
| TMB with errors and response with misclassification  e ~ Extreme (0, ${1.5}^{2}$)  $\left( \eta,\delta\right)=\left( 0.70,0.95 \right)$  Naive estimator | $\lambda$ | 0.918 | -0.082 | 0.044 | 0.053 |
|  | $\beta$ | 0.586 | -0.414 | 0.106 | 0.128 |
|  | $\beta_{m}$ | -0.180 | 0.220 | 0.025 | 0.031 |
|  | $\alpha$ | -1.104 | -0.304 | 0.191 | 0.187 |
|  | $\alpha_{m}$ | 0.107 | -0.293 | 0.046 | 0.043 |
|  | $\sigma_{b}$ | 0.446 | -0.054 | 0.086 | 0.118 |
| TMB with errors and response with misclassification  e ~ Extreme (0, ${1.5}^{2}$)  $\left( \eta,\delta\right)=\left( 0.70,0.95 \right)$  Bayesian estimator | $\lambda$ | 1.015 | 0.015 | 0.094 | 0.108 |
|  | $\beta$ | 0.925 | -0.075 | 0.288 | 0.339 |
|  | $\beta_{m}$ | -0.414 | -0.014 | 0.138 | 0.166 |
|  | $\alpha$ | -0.785 | 0.015 | 0.626 | 0.385 |
|  | $\alpha_{m}$ | 0.380 | -0.020 | 0.306 | 0.193 |
|  | $\sigma_{b}$ | 0.473 | -0.027 | 0.156 | 0.247 |
|  | $\eta$ | 0.735 | 0.035 | 0.048 | 0.016 |
|  | $\delta$ | 0.963 | 0.013 | 0.021 | 0.004 |
| TMB with errors and response with misclassification  e ~ Extreme (0, ${1.5}^{2}$)  $\left( \eta,\delta\right)=\left( 0.75,0.98 \right)$  Naive estimator | $\lambda$ | 0.917 | -0.083 | 0.043 | 0.049 |
|  | $\beta$ | 0.578 | -0.422 | 0.105 | 0.120 |
|  | $\beta_{m}$ | -0.180 | 0.220 | 0.025 | 0.029 |
|  | $\alpha$ | -1.084 | -0.284 | 0.190 | 0.184 |
|  | $\alpha_{m}$ | 0.123 | -0.277 | 0.046 | 0.046 |
|  | $\sigma_{b}$ | 0.438 | -0.062 | 0.084 | 0.128 |
| TMB with errors and response with misclassification  e ~ Extreme (0, ${1.5}^{2}$)  $\left( \eta,\delta\right)=\left( 0.75,0.98 \right)$  Bayesian estimator | $\lambda$ | 1.016 | 0.016 | 0.093 | 0.119 |
|  | $\beta$ | 0.928 | -0.072 | 0.286 | 0.339 |
|  | $\beta_{m}$ | -0.402 | -0.002 | 0.132 | 0.166 |
|  | $\alpha$ | -0.744 | 0.056 | 0.642 | 0.413 |
|  | $\alpha_{m}$ | 0.406 | 0.006 | 0.306 | 0.192 |
|  | $\sigma_{b}$ | 0.481 | -0.019 | 0.151 | 0.266 |
|  | $\eta$ | 0.737 | -0.013 | 0.048 | 0.016 |
|  | $\delta$ | 0.960 | -0.020 | 0.022 | 0.005 |
