# Supplementary material for: TMBocelot: an omnibus statistical control model optimizing the TMB thresholds with systematic measurement errors
Source: Front Immunol. 2025 Jan 20;15:1514295. doi: 10.3389/fimmu.2024.1514295 (PMC11788372; doi:10.3389/fimmu.2024.1514295)
Supplement: Supplementary Material — The technical appendices for TMBocelot. [file DataSheet1.docx]

Supplementary Material

TMBocelot: an Omnibus statistical Control modeL Optimizing the TMB Thresholds with systematic measurement errors

**Xin Lai**^1†^**, Shaoliang Wang**^1†^**, Xuanping Zhang**^1^**, Xiaoyan Zhu**^1^**, Yuqian Liu**^1^**, Zhili Chang**^3^**, Xiaonan Wang**^3^**, Yang Shao**^3, 4^**, Jiayin Wang**^1*^**, Yixuan Wang**^2*^

^1^ School of Computer Science and Technology, Faculty of Electronics and Information Engineering, Xi’an Jiaotong University, Xi’an, Shaanxi, China

^2^ Department of Biomedical Engineering, College of Automation Engineering, Nanjing University of Aeronautics and Astronautics, Nanjing, China

^3^ Nanjing Geneseeq Technology Inc., Nanjing, Jiangsu, China

^4^ School of Public Health, Nanjing Medical University, Nanjing, Jiangsu, China

^†^These authors have contributed equally to this work and share first authorship.

*** Correspondence:**

Jiayin Wang
[wangjiayin@mail.xjtu.edu.cn](mailto:wangjiayin@mail.xjtu.edu.cn)
Yixuan Wang

wangyixuan@nuaa.edu.cn

# Bias Arising from TMB Error and Response Misclassification

Based on past studies [1], we illustrate the negative effects of TMB error again and add the effect of Response misclassification. When there is no measurement error, the score functions are unbiased. Specifically, define $\theta^{T}=\left[ \theta_{R}^{T},\theta_{T}^{T},\theta_{b} \right]$, $\theta_{R}=\left[ \alpha_{Z}^{T},\alpha_{m} \right]^{T}$, $\theta_{T}=\left[ \lambda,\beta_{Z}^{T},\beta_{m} \right]$, $\theta_{b}=\sigma_{b}$, then we have:

$$\Psi_{i}\left( R_{i},T_{i},\Delta_{i},Z_{i},TMB_{i};\Theta\right)=\frac{\partial{\tilde{\mathcal{l}}}_{i}\left( \theta,\hat{b}_{i} \right)}{\partial\theta^{T}}=\Psi_{R,i}\left( \theta\right)+\Psi_{T.i}\left( \theta\right)+\Psi_{b.i}\left( \theta\right)$$

$$\Psi_{i}\left( \theta_{R} \right)=\Psi_{R,i}\left( \theta_{R} \right)+\Psi_{b,i}\left( \theta_{R} \right)=\left\{ R_{i}-F(\alpha_{z}^{T}Z_{i}+\alpha_{m}{TMB}_{i}+\hat{b}_{i}) \right.\left. -\frac{1}{2}\frac{\exp\left( \alpha_{z}^{T}\mathbf{Z}_{i}+\alpha_{m}{TMB}_{i}+\hat{b}_{i} \right)\left\{ 1-exp\left( \alpha_{z}^{T}\mathbf{Z}_{i}+\alpha_{m}{TMB}_{i}+\hat{b}_{i} \right) \right\}}{\left| k^{''}\left( \hat{b}_{i};\theta\right) \right|\left\{ 1+exp\left( \alpha_{z}^{T}\mathbf{Z}_{i}+\alpha_{m}{TMB}_{i}+\hat{b}_{i} \right) \right\}^{3}} \right\}\left( \frac{\mathbf{Z}_{i}}{{TMB}_{i}} \right)$$

$$\Psi_{i}\left( \lambda\right)=\Psi_{T,i}\left( \lambda\right)+\Psi_{b,i}\left( \lambda\right)$$

$$=\Delta_{i}\left( \lambda^{-1}+logT_{i} \right)-T_{i}^{\lambda}\log T_{i}\exp\left( \beta_{z}^{T}\mathbf{Z}_{i}+\beta_{m}{TMB}_{i}+\hat{b}_{i} \right)+\frac{1}{2}\frac{T_{i}^{\lambda}\log T_{i}\exp\left( \beta_{z}^{T}\mathbf{Z}_{i}+\beta_{m}{TMB}_{i}+\hat{b}_{i} \right)}{\left| k^{''}\left( \hat{b}_{i};\theta\right) \right|}$$

$$\Psi_{i}\left( \beta_{z}^{T},\beta_{m} \right)=\Psi_{T,i}\left( \beta_{z}^{T},\beta_{m} \right)+\Psi_{b,i}\left( \beta_{z}^{T},\beta_{m} \right)$$

$$=\left\{ \Delta_{i}-T_{i}^{\lambda}exp(\beta_{z}^{T}\mathbf{Z}_{i}+\beta_{m}{TMB}_{i}+\hat{b}_{i}) \right.+\frac{1}{2}\left. \frac{T_{i}^{\lambda}\exp\left( \beta_{z}^{T}Z_{i}+\beta_{m}{TMB}_{i}+\hat{b}_{i} \right)}{\left| k^{''}\left( \hat{b}_{i};\theta\right) \right|} \right\}\left( \frac{Z_{i}}{{TMB}_{i}} \right)$$

$$\Psi_{i}\left( \sigma_{b} \right)=\Psi_{b,i}\left( \sigma_{b} \right)=-\sigma_{b}^{-1}+\hat{b}_{1}^{2}\cdot\sigma_{b}^{-3}+\left( \sigma_{b}\left| k^{''}\left( \hat{b}_{i};\theta\right) \right| \right)^{-1}$$

where the score function $\Psi$ is conditionally unbiased for the approximate likelihood:

$$E\left\{ \Psi\left( R_{i},T_{i},\Delta_{i},Z_{i},{TMB}_{i};\Theta\right) \right\}=0, i=1,\ldots,n$$

When under TMB errors, that is ${{TMB}_{i}}^{*}={TMB}_{i}+e_{i}$, the unbiasedness of score function $\Psi$ is destroyed, that is $E\left\{ \Psi\left( {{TMB}_{i}}^{*};\Theta\right) \right\}=E\left\{ \Psi\left( {TMB}_{i}+e_{i};\Theta\right) \right\}\neq0$, which was seen in both the part of survival function and response score function. For example,

$$\boldsymbol{E}\left\{ \Delta_{i}-T_{i}^{\lambda}exp(\beta_{z}^{T}\mathbf{Z}_{i}+\beta_{m}{{TMB}_{i}}^{*}+\hat{b}_{i}) \right\}$$

$$\boldsymbol{=E}\left\{ \Delta_{i}-T_{i}^{\lambda}exp(\beta_{z}^{T}\mathbf{Z}_{i}+\beta_{m}{TMB}_{i}+\beta_{m}e_{i}+\hat{b}_{i}) \right\}$$

$$\boldsymbol{=}\Delta_{i}-T_{i}^{\lambda}E\left\{ \exp\left( \beta_{z}^{T}\mathbf{Z}_{i}+\beta_{m}{TMB}_{i}+\beta_{m}e_{i}+\hat{b}_{i} \right) \right\}$$

$$=\Delta_{i}-T_{i}^{\lambda}\exp\left( \beta_{z}^{T}\mathbf{Z}_{i}+\beta_{m}TMB_{i}+\hat{b}_{i} \right)E\left\{ \exp\left( \beta_{m}e_{i} \right) \right\}$$

$$E\left\{ F\left( \alpha_{z}^{T}\mathbf{Z}_{i}+\alpha_{m}{TMB}_{i}+\alpha_{m}e_{i}+\hat{b}_{i} \right) \right\}$$

$$=\int_{-\infty}^{+\infty} F\left( \alpha_{z}^{T}\mathbf{Z}_{i}+\alpha_{m}{TMB}_{i}+\alpha_{m}e_{i}+\hat{b}_{i} \right)p\left( e_{i} \right)de_{i}$$

where additional term $E\left\{ \exp\left( \beta_{m}e_{i} \right) \right\}$ and $p\left( e_{i} \right)$ on the scoring function is generated by the measurement error, leading the naive estimator to be biased apparently.

Similarly, when under Response misclassification, the unbiasedness of score function $\Psi$ is also destroyed, that is $E\left\{ \Psi\left( {R_{i}}^{*};\Theta\right) \right\}=E\left\{ \Psi\left( R_{i}+e_{i};\Theta\right) \right\}\neq0$. Specifically, we can find that:

$$E\left( {R_{i}}^{*}|R_{i} \right)=E\left( R_{i}+e_{i}|R_{i} \right)=\eta^{R_{i}}\left( 1-\delta\right)^{1-R_{i}}\neq R_{i}$$

where the presence of $e_{i}$ cause $\eta\neq1$ or $\delta\neq1$. And due to $E\left( R_{i}+e_{i}|R_{i} \right)\neq R_{i}$, the naive estimator is biased apparently.

The presence of $e_{i}$ on TMB or Response biased the score function, leading to a bias in naive estimator. **Figure S1** illustrate the impact of pairwise error in immunotherapy.


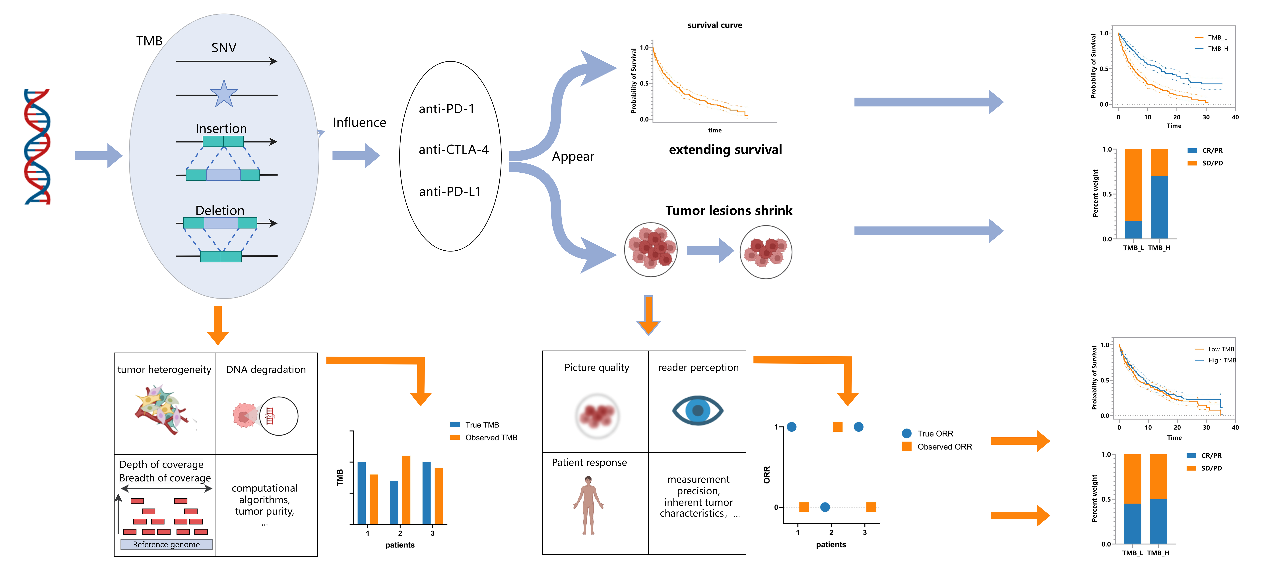


Figure S1 The impact of pairwise error in immunotherapy

# Model about Robust correction method for unspecified TMB error

The following part is a detailed introduction of the various parameter settings in the robust correction method for unspecified TMB error of the article.

Specifically, for TMB errors, it can be expressed equivalently as:

$$f_{e}\left( e \right)=\sum_{i}^{K} \pi_{i}N\left( {\mu_{e}}_{i},{{\sigma_{e}}_{i}}^{2} \right)$$

where $f_{e}\left( e \right)$ is a Gaussian Mixture distribution and $K$ was determined by the data itself, which can approximate any distribution in theory. And the above model can be extended as follows:

$$e_{i}\sim N\left( {\mu_{e}}_{i},{{\sigma_{e}}_{i}}^{2} \right)$$

$${\mu_{e}}_{i},{{\sigma_{e}}_{i}}^{2}\sim G$$

$$G\sim DP\left( M_{e}, G_{e} \right)$$

$$M_{e}\sim gamma\left( v_{1},v_{2} \right)$$

where $v_{1},v_{2}=0.001$ are fixed constants. Marginalize G, and we can derive that:

$${\mu_{e}}_{i},{{\sigma_{e}}_{i}}^{2}|{\mu_{e}}_{-i},{{\sigma_{e}}_{-i}}^{2}\sim\left\{ \begin{aligned} \frac{1}{i-1+M_{e}}\sum_{j}^{-i} \delta\left( {\mu_{e}}_{j},{{\sigma_{e}}_{j}}^{2} \right) {\mu_{e}}_{i},{{\sigma_{e}}_{i}}^{2}\in{\mu_{e}}_{-i},{{\sigma_{e}}_{-i}}^{2} \\ \frac{M_{e}}{i-1+M_{e}} G_{e} {\mu_{e}}_{i},{{\sigma_{e}}_{i}}^{2}\notin{\mu_{e}}_{-i},{{\sigma_{e}}_{-i}}^{2} \end{aligned} \right.$$

where $-i$ represents $\left\{ 1, 2, \ldots, i-1, i+1,\ldots,n \right\}$, $\delta\left( \cdot\right)$ the indicator function.

Hence, $G_{e}$ can be denoted as:

$$G_{e}\left\{ \begin{aligned} {\mu_{e}}_{i}\sim N\left( a, {g_{1}\cdot{\sigma_{e}}_{i}}^{2} \right) \\ {{\sigma_{e}}_{i}}^{-2}\sim gamma(s_{1},s_{2}) \end{aligned} \right.$$

where $s_{1},s_{2},g_{1}$ are fixed constants and $0=E\left( e_{i} \right)=E\left\{ E\left( e_{i}|{\mu_{e}}_{i} \right) \right\}=E\left( {\mu_{e}}_{i} \right)=a$.

In addition, we can find $0<\mathrm{var}\left( e_{i} \right)<\mathrm{var}\left( {{TMB}_{i}}^{*} \right)$ and $\mathrm{var}\left( e_{i} \right)=E\left\{ \mathrm{var}\left( e_{i} \right)|{{\sigma_{e}}_{i}}^{2} \right\}=E\left( {{\sigma_{e}}_{i}}^{2} \right)$. In order to improve the sampling efficiency, we set $E\left( {{\sigma_{e}}_{i}}^{2} \right)={\mathrm{var}\left( {TMB}^{*} \right)}/2$, $\mathrm{var}\left( {{\sigma_{e}}_{i}}^{2} \right)=\left( \mathrm{var}\left( {TMB}^{*} \right) \right)^{2}$as approximate prior parameters. So, we set $s_{1}=9/4$ and $s_{2}=5/8* \mathrm{var}\left( {TMB}^{*} \right)$.

Similarly, for TMB true values, we denoted that:

$$f_{TMB}\left( TMB \right)=\sum_{i}^{K} \pi_{i}N\left( {\mu_{TMB}}_{i},{{\sigma_{TMB}}_{i}}^{2} \right)$$

$${TMB}_{i}\sim N\left( {\mu_{TMB}}_{i},{{\sigma_{TMB}}_{i}}^{2} \right)$$

$${\mu_{TMB}}_{i},{{\sigma_{TMB}}_{i}}^{2}\sim G$$

$$G\sim DP\left( M_{TMB}, G_{TMB} \right)$$

$$M_{TMB}\sim gamma\left( v_{3},v_{4} \right)$$

$$G_{TMB}\left\{ \begin{aligned} {\mu_{TMB}}_{i}\sim N\left( b,g_{2}\cdot{{\sigma_{TMB}}_{i}}^{2} \right) \\ {{\sigma_{TMB}}_{i}}^{-2}\sim gamma(s_{3},s_{4}) \end{aligned} \right.$$

where $v_{3},v_{4},g_{2},s_{3},s_{4}$ are fixed constants and $E\left( {{TMB}_{i}}^{*} \right)=E\left( {TMB}_{i} \right)=E\left\{ E\left( {TMB}_{i}|{\mu_{TMB}}_{i} \right) \right\}=E\left( {\mu_{TMB}}_{i} \right)=b$. $v_{3},v_{4}=0.001$, $g_{2}=0.5$, $s_{3}=9/4$ and $s_{4}=5/8* \mathrm{var}\left( {TMB}^{*} \right)$.

The above formulas form the core of robust TMB measurement error model, with more details in the section3.

# Estimation method

The parameter estimation method we report is based on the MCMC algorithm, and its overall sampling algorithm is based on Gibbs sampling [2], which is an algorithm that alternately samples the parameters to achieve convergence. For a specific parameter, we sample its fully conditional distribution, that is, the posterior distribution given the remaining parameters.

The primary mixed-endpoint model can be expressed as two parts. Specifically, for patient $i(i=1,2,\ldots,n)$,

the logistical regression sub-model for the ORR endpoint can be expressed as:

$$\begin{aligned} \mathrm{logit}\left( R_{i}|Z_{i},{TMB}_{i},b_{i};\theta\right)={\alpha_{z}}^{T}Z_{i}+\alpha_{m}{TMB}_{i}+b_{i}\#\left（ S1 \right） \end{aligned}$$

the Cox PH regression sub-model for the TTE endpoint can be expressed as:

$$h_{i}\left( t|Z_{i},{TMB}_{i},b_{i};\theta\right)=h_{0}\left( t \right)\exp\left( {\beta_{z}}^{T}Z_{i}+\beta_{m}{TMB}_{i}+b_{i} \right)$$

$$S_{i}\left( t|Z_{i},{TMB}_{i},b_{i};\theta\right)=\exp\left\{ -\int_{0}^{t} h_{0}\left( s \right)\exp\left( {\beta_{z}}^{T}Z_{i}+\beta_{m}{TMB}_{i}+b_{i} \right)ds \right\}$$

$$=\exp\left\{ -H_{0}\left( t \right)\exp\left( {\beta_{z}}^{T}Z_{i}+\beta_{m}{TMB}_{i}+b_{i} \right) \right\}$$

$$h_{0}\left( t \right)=\lambda t^{\lambda-1}$$

$$\begin{aligned} \#\left（ S2 \right） \end{aligned}$$

## A mixed-endpoint model without measurement error of TMB and misclassification of endpoints

The specific estimation procedure can be described as **Algorithm 1.**

1) Assign prior distributions:

$$\alpha_{z},\alpha_{m},\beta_{z},\beta_{m}\sim N\left( 0,{10}^{2} \right)$$

$$\lambda\sim gamma\left( 0.001,0.001 \right)$$

$${\sigma_{b}}^{-2}\sim gamma\left( 0.001,0.001 \right)$$

$$b_{i}\sim N\left( 0，{\sigma_{b}}^{2} \right)$$

2) Compute posterior distributions:

$$\alpha_{z},\alpha_{m}|\ldots\propto Prior\left( \alpha_{z},\alpha_{m} \right)*L\left( R|\alpha_{z},\alpha_{m},\ldots\right)$$

$$=Prior\left( \alpha_{z},\alpha_{m} \right)*\prod_{i} p\left( R_{i}|b_{i},{TMB}_{i};\theta\right)$$

$$\beta_{z},\beta_{m},\lambda|\ldots\propto Prior\left( \beta_{z},\beta_{m},\lambda\right)*L\left( T,\Delta|\beta_{z},\beta_{m},\lambda,\ldots\right)$$

$$=Prior\left( \beta_{z},\beta_{m},\lambda\right)*\prod_{i} p\left( T_{i},\Delta_{i}|b_{i},{TMB}_{i};\theta\right)$$

$$b_{i}|\ldots\propto Prior\left( b_{i} \right)*p\left( R_{i}|b_{i},{TMB}_{i};\theta\right)p\left( T_{i},\Delta_{i}|b_{i},{TMB}_{i};\theta\right)$$

$${\sigma_{b}}^{-2}|\ldots\propto Prior\left( {\sigma_{b}}^{-2} \right)\prod_{i} p\left( b_{i} \right)$$

where

$$p\left( R_{i}|b_{i},{TMB}_{i};\theta\right)={F\left( {\alpha_{z}}^{T}Z_{i}+\alpha_{m}{TMB}_{i}+b_{i} \right)}^{R_{i}}$$

$${\cdot\left\{ 1-F\left( {\alpha_{z}}^{T}Z_{i}+\alpha_{m}{TMB}_{i}+b_{i} \right) \right\}}^{1-R_{i}}$$

$$F\left( v \right)=\left( 1+e^{-v} \right)^{-1}$$

$$p\left( T_{i},\Delta_{i}|b_{i};\theta\right)={h_{i}\left( T_{i}|b_{i};\theta\right)}^{\Delta_{i}}S_{i}\left( T_{i}|b_{i};\theta\right)$$

$$p\left( b_{i} \right)=Prior\left( b_{i} \right)$$

3) Sample by Sample algorithm:

For parameters $\alpha_{z},\alpha_{m},\beta_{z},\beta_{m}, \lambda$ and $b_{i}$, we used adaptive Metropolis algorithm [3] sampling;

For parameters ${\sigma_{b}}^{2}$, we used conjugate sampling.

4) Calculate parameter estimates according to Monte Carlo:

$$\hat{\theta}\approx\frac{1}{K}\sum_{k=1}^{K} \theta_{k}^{'}$$

where $\theta_{k}^{'}$ is the sample value and K is the number of samplings.

## A mixed-endpoint model just considering TMB errors without side information

Then the **algorithm 1.2** can be supplemented on the basis of **algorithm 1.1**. And the addition is described as:

1) Assign prior distributions:

$$e_{i}\sim N\left( {\mu_{e}}_{i},{{\sigma_{e}}_{i}}^{2} \right)$$

$${\mu_{e}}_{i},{{\sigma_{e}}_{i}}^{2}|{\mu_{e}}_{-i},{{\sigma_{e}}_{-i}}^{2}\propto$$

$$\left\{ \begin{aligned} \frac{1}{i-1+M_{e}}\sum_{j}^{-i} \delta\left( {\mu_{e}}_{j},{{\sigma_{e}}_{j}}^{2} \right) {\mu_{e}}_{i},{{\sigma_{e}}_{i}}^{2}\in{\mu_{e}}_{-i},{{\sigma_{e}}_{-i}}^{2} \\ \frac{M_{e}}{i-1+M_{e}} G_{e} {\mu_{e}}_{i},{{\sigma_{e}}_{i}}^{2}\notin{\mu_{e}}_{-i},{{\sigma_{e}}_{-i}}^{2} \end{aligned} \right.$$

$$M_{e}\sim gamma\left( v_{1},v_{2} \right)$$

$${TMB}_{i}\sim N\left( {\mu_{TMB}}_{i},{{\sigma_{TMB}}_{i}}^{2} \right)$$

$${\mu_{TMB}}_{i},{{\sigma_{TMB}}_{i}}^{2}|{\mu_{TMB}}_{-i},{{\sigma_{TMB}}_{-i}}^{2}\propto$$

$$\left\{ \begin{aligned} \frac{1}{i-1+M_{TMB}}\sum_{j}^{-i} \delta\left( {\mu_{TMB}}_{i},{{\sigma_{TMB}}_{i}}^{2} \right){\mu_{TMB}}_{i},{{\sigma_{TMB}}_{i}}^{2}\in{\mu_{TMB}}_{-i},{{\sigma_{TMB}}_{-i}}^{2} \\ \frac{M_{TMB}}{i-1+M_{TMB}} G_{TMB} {\mu_{TMB}}_{i},{{\sigma_{TMB}}_{i}}^{2}\notin{\mu_{TMB}}_{-i},{{\sigma_{TMB}}_{-i}}^{2} \end{aligned} \right.$$

$$M_{TMB}\sim gamma\left( v_{3},v_{4} \right)$$

2) Compute posterior distributions:

$${TMB}_{i}|\ldots\propto prior\left( {TMB}_{i} \right)*p\left( R_{i}|b_{i},{TMB}_{i};\theta\right)p\left( T_{i},\Delta_{i}|b_{i},{TMB}_{i};\theta\right)p\left( {{TMB}_{i}}^{*}|{TMB}_{i} \right)$$

$${\mu_{TMB}}_{i},{{\sigma_{TMB}}_{i}}^{2}|\ldots\propto prior\left( {\mu_{TMB}}_{i},{{\sigma_{TMB}}_{i}}^{2} \right)*p\left( {TMB}_{i}|{\mu_{TMB}}_{i},{{\sigma_{TMB}}_{i}}^{2} \right)$$

$${\mu_{e}}_{i},{{\sigma_{e}}_{i}}^{2}|\ldots\propto prior\left( {\mu_{e}}_{i},{{\sigma_{e}}_{i}}^{2} \right)*p\left( e_{i}|{\mu_{e}}_{i},{{\sigma_{e}}_{i}}^{2} \right)$$

$$M_{TMB}|\ldots\propto prior\left( M_{TMB} \right)p\left( k|M_{TMB} \right)$$

$$M_{e}|\ldots\propto prior\left( M_{e} \right)p\left( k|M_{e} \right)$$

where $k$ denotes the number of categories and

$$p\left( k|M_{TMB} \right)\propto\frac{{M_{TMB}}^{k}\Gamma\left( M_{TMB} \right)}{\Gamma\left( M_{TMB}+n \right)}={M_{TMB}}^{k}\frac{M_{TMB}+n}{M_{TMB}\Gamma\left( n \right)}\int_{0}^{1} \varphi^{M_{TMB}}\left( 1-\varphi\right)^{n-1}d\varphi$$

$$p\left( k|M_{e} \right)\propto\frac{{M_{e}}^{k}\Gamma\left( M_{e} \right)}{\Gamma\left( M_{e}+n \right)}={M_{e}}^{k}\frac{M_{e}+n}{M_{e}\Gamma\left( n \right)}\int_{0}^{1} \varphi^{M_{e}}\left( 1-\varphi\right)^{n-1}d\varphi$$

$$p\left( {{TMB}_{i}}^{*}|{TMB}_{i} \right)=prior\left( e_{i} \right)=\sum_{i=1}^{K} \pi_{i}N\left( {\mu_{e}}_{i},{{\sigma_{e}}_{i}}^{2} \right)$$

$$prior\left( {TMB}_{i} \right)=\sum_{i=1}^{K} \pi_{i}N\left( {\mu_{TMB}}_{i},{{\sigma_{TMB}}_{i}}^{2} \right)$$

3) Sample by Sample algorithm:

For parameters ${TMB}_{i}$, we used adaptive Metropolis sampling and $e_{i}={{TMB}_{i}}^{*}-{TMB}_{i}$.

For parameters $M_{TMB}$ and $M_{e}$, we used conjugate sampling with a latent variable $\varphi$ [4]. Specifically, the posterior distribution of $M_{e}$ can be denoted as:

$$p\left( M_{e}|\ldots\right)\propto prior\left( M_{e} \right){M_{e}}^{k-1}\left( M_{e}+n \right)\int_{0}^{1} \varphi^{M_{e}}\left( 1-\varphi\right)^{n-1}d\varphi$$

which implies that $p\left( M_{e}|\ldots\right)$ is the marginal distribution from a joint for $M_{e}$ and a continuous quantity $\varphi$ such that

$$p\left( M_{e},\varphi|\ldots\right)\propto prior\left( M_{e} \right){M_{e}}^{k-1}\left( M_{e}+n \right)\varphi^{M_{e}}\left( 1-\varphi\right)^{n-1}$$

where $\varphi$ is a latent variable and $0<\varphi<1$.

Hence, we have conditional posteriors $p\left( M_{e}|\varphi,\ldots\right)$ and $p\left( \varphi|M_{e},\ldots\right)$, determined as follows:

$$p\left( \varphi|M_{e},\ldots\right)\sim Beta\left( M_{e}+1,n \right)$$

$$p\left( M_{e}|\varphi,\ldots\right)\propto prior\left( M_{e} \right){M_{e}}^{k-1}\left( M_{e}+n \right)\varphi^{M_{e}}$$

$$={M_{e}}^{v_{1}+k-2}\left( M_{e}+n \right)e^{-M_{e}\left( v_{2}-log(\varphi\right)}$$

$$={M_{e}}^{v_{1}+k-1}e^{-M_{e}\left( v_{2}-log(\varphi) \right)}+n{M_{e}}^{v_{1}+k-2}e^{-M_{e}\left( v_{2}-log(\varphi) \right)}$$

where the latter reduces easily to a mixture of two gamma densities, that is,

$$p\left( M_{e}|\varphi,\ldots\right)\propto\pi_{\varphi}gamma\left( v_{1}+k,v_{2}-\log\left( \varphi\right) \right)+\left( 1-\pi_{\varphi} \right)gamma\left( v_{1}+k-1,v_{2}-\log\left( \varphi\right) \right)$$

where weights $\pi_{\varphi}$ defined by ${\pi_{\varphi}}/\left( 1-\pi_{\varphi} \right)=\left( v_{1}+k-1 \right)/\left\{ n\left( v_{2}-\log\left( \varphi\right) \right) \right\}$.

So, for parameter $M_{e}$, We can sample $p\left( \varphi|M_{e},\ldots\right)$ and $p\left( M_{e}|\varphi,\ldots\right)$ alternately, and it’s similar for parameter $M_{TMB}.$

For parameters ${\mu_{e}}_{i},{{\sigma_{e}}_{i}}^{2}$ and ${\mu_{TMB}}_{i},{{\sigma_{TMB}}_{i}}^{2}$, we used Metropolis-Hastings algorithm for DP mixture [5] sampling. For improving the efficacy of sampling, we introduced latent variables $c_{i}$, which denote the category of $e_{i}$ or ${TMB}_{i}$.

Specifically, for ${\mu_{e}}_{i},{{\sigma_{e}}_{i}}^{2}$, we divided the sampling process into two steps.

i) sampling latent variables $c_{i}$

$$c_{i}|c_{-i}\propto\left\{ \begin{aligned} \frac{1}{i-1+M_{e}}\sum_{j}^{-i} \delta\left( c_{j} \right), c_{i}\in c_{-i} \\ \frac{M_{e}}{i-1+M_{e}}, c_{i}\notin c_{-i} \end{aligned} \right.$$

with probability of acceptance

$$\alpha=\min\left\{ \frac{p\left( e_{i}|\left( {\mu_{e}}_{i},{{\sigma_{e}}_{i}}^{2} \right)^{new} \right)}{p\left( e_{i}|\left( {\mu_{e}}_{i},{{\sigma_{e}}_{i}}^{2} \right)^{old} \right)},1 \right\}$$

where

$$\left( {\mu_{e}}_{i},{{\sigma_{e}}_{i}}^{2} \right)^{new}=\left\{ \begin{aligned} {\mu_{e}}_{j},{{\sigma_{e}}_{j}}^{2} c_{i}=c_{j}, j\in-i \\ \mu,\sigma^{2}\sim G_{e} c_{i}\notin c_{-i} \end{aligned} \right.$$

ii) sampling parameters ${\mu_{e}}_{i},{{\sigma_{e}}_{i}}^{2}$ belonging to the same category

$${\mu_{e}}_{c},{{\sigma_{e}}_{c}}^{2}\propto G_{e}\left( {\mu_{e}}_{c},{{\sigma_{e}}_{c}}^{2} \right)\prod_{i}^{c} p\left( e_{i}|{\mu_{e}}_{i},{{\sigma_{e}}_{i}}^{2} \right)$$

And it’s similar for parameters ${\mu_{TMB}}_{i},{{\sigma_{TMB}}_{i}}^{2}$.

## A mixed-endpoint model just considering misclassification of endpoints

When considering misclassification of tumor response, new parameters was introduced, which can be denoted as:

$$\eta=P\left( R_{i}=1|Y_{i}=1 \right)$$

$$\delta=P\left( R_{i}=0|Y_{i}=0 \right)$$

Then **Eq. S1** was modified as

$$\mathrm{logit}\left( Y_{i}|Z_{i},{TMB}_{i},b_{i};\theta\right)={\alpha_{z}}^{T}Z_{i}+\alpha_{m}{TMB}_{i}+b_{i}$$

And the corresponding model for the observed ORR endpoint is formulated as:

$$P\left( R_{i}=1|{TMB}_{i},b_{i};\theta\right)$$

$$=P\left( Y_{i}=1 \right)P\left( R_{i}=1|Y_{i}=1 \right)+P\left( Y_{i}=0 \right)\left[ 1-P\left( R_{i}=0|Y_{i}=0 \right) \right]$$

$$=\eta P\left( Y_{i}=1 \right)+\left( 1-\delta\right)\left( 1-P\left( Y_{i}=1 \right) \right)$$

So, **algorithm 1.3** can be supplemented on the basis of **algorithm 1.1**, but with a formula was modified as:

$$p\left( R_{i}|b_{i},{TMB}_{i};\theta\right)={P\left( R_{i}=1|{TMB}_{i},b_{i};\theta\right)}^{R_{i}}\left( 1-P\left( R_{i}=1|{TMB}_{i},b_{i};\theta\right) \right)^{1-R_{i}}$$

And the addition is described as:

1) Assign prior distributions:

$$\eta\sim Beta\left( \epsilon_{1},\epsilon_{2} \right)$$

$$\delta\sim Beta\left( \epsilon_{3},\epsilon_{4} \right)$$

2) Compute posterior distributions:

$$\eta,\delta|\ldots\sim prior\left( \eta,\delta\right)L\left( R|\eta，\theta，\ldots\right)$$

$$= prior\left( \eta,\delta\right)\prod_{i} p\left( R_{i}|b_{i},{TMB}_{i};\theta\right)$$

where

$$p\left( R_{i}|b_{i},{TMB}_{i};\theta\right)={U\left( {\alpha_{z}}^{T}Z_{i}+\alpha_{m}{TMB}_{i}+b_{i} \right)}^{R_{i}}$$

$${\cdot\left\{ 1-U\left( {\alpha_{z}}^{T}Z_{i}+\alpha_{m}{TMB}_{i}+b_{i} \right) \right\}}^{1-R_{i}}$$

$$U\left( v \right)=\eta\left( 1+e^{-v} \right)^{-1}+\left( 1-\delta\right)\left( 1+e^{v} \right)^{-1}$$

3) Sample by Sample algorithm:

For parameters $\eta,\delta$, we used adaptive Metropolis-Hastings algorithm sampling.

## A mixed-endpoint model jointly considering TMB errors and misclassification of endpoints

Algorithm 1.4 is a combination of Algorithm 1.2 and Algorithm 1.3.

# Reference

Wang Y, Lai X, Wang J, et al. A Joint Model Considering Measurement Errors for Optimally Identifying Tumor Mutation Burden Threshold. *Front Genet* 2022;13:915839.

Casella G, George E I. Explaining the Gibbs sampler. *The American Statistician* 1992, 46(3): 167-174.

Haario H, Saksman E, Tamminen J. An adaptive Metropolis algorithm. *Bernoulli* 2001: 223-242.

Escobar M D, West M. Bayesian density estimation and inference using mixtures. *Journal of the american statistical association* 1995, 90(430): 577-588.

Neal R M. Markov chain sampling methods for Dirichlet process mixture models. Journal of computational and graphical statistics 2000;9(2):249-265.
